# Cell death-induced immunogenicity enhances chemoimmunotherapeutic response by converting immune-excluded into T-cell inflamed bladder tumors

**DOI:** 10.1038/s41467-022-29026-9

**Published:** 2022-03-28

**Authors:** Fotis Nikolos, Kazukuni Hayashi, Xen Ping Hoi, Mark Ellie Alonzo, Qianxing Mo, Armine Kasabyan, Hideki Furuya, Jane Trepel, Dolores Di Vizio, Jlenia Guarnerio, Dan Theodorescu, Charles Rosser, Andrea Apolo, Matthew Galsky, Keith Syson Chan

**Affiliations:** 1 Samuel Oschin Cancer Center, Cedars-Sinai Medical Center, Los Angeles, CA 90048 USA; 2 Department of Pathology and Laboratory Medicine, Cedars-Sinai Medical Center, Los Angeles, CA 90048 USA; 3 Graduate Program in Translational Biology and Molecular Medicine, Baylor College of Medicine, Houston, TX 77030 USA; 4 Department of Biomedical Sciences, Cedars-Sinai Medical Center, Los Angeles, CA USA; 5 H. Lee Moffitt Cancer Center and Research Institute, Tampa, FL USA; 6 Department of Surgery (Urology), Cedars-Sinai Medical Center, Los Angeles, CA USA; 7 Center for Cancer Research, National Cancer Institute, Bethesda, MD USA; 8 Tisch Cancer Institute, Icahn School of Medicine at Mount Sinai, New York, NY USA

**Keywords:** Cancer immunotherapy, Chemotherapy, Bladder cancer

## Abstract

Chemoimmunotherapy has recently failed to demonstrate significant clinical benefit in advanced bladder cancer patients; and the mechanism(s) underlying such suboptimal response remain elusive. To date, most studies have focused on tumor-intrinsic properties that render them “immune-excluded”. Here, we explore an alternative, drug-induced mechanism that impedes therapeutic response via disrupting the onset of immunogenic cell death. Using two immune-excluded syngeneic mouse models of muscle-invasive bladder cancer (MIBC), we show that platinum-based chemotherapy diminishes CD8+ T cell tumor infiltration and constraines their antitumoral activity, despite expression of activation markers IFNγ and granzyme B. Mechanistically, chemotherapy induces the release of prostaglandin E_2_ (PGE_2_) from dying cancer cells, which is an inhibitory damage-associated molecular pattern (iDAMP) that hinderes dendritic cell maturation. Upon pharmaceutical blockade of PGE_2_ release, CD8+ T cells become tumoricidal and display an intraepithelial-infiltrating (or inflamed) pattern. This “iDAMP blockade” approach synergizes with chemotherapy and sensitizes bladder tumors towards anti-PD1 immune checkpoint inhibitor therapy. These findings provide a compelling rationale to evaluate this drug combination in future clinical trials.

## Abstract (web summary)

Chemoimmunotherapy recently failed to improve objective response for patients with advanced muscle-invasive bladder cancer (MIBC). Here using two murine models of immune-excluded MIBC, the authors show that resistance to chemoimmunotherapy can be overcome by blocking the COX-2/prostaglandin E2 axis, reinvigorating anti-tumor immune responses.

## Introduction

Bladder cancer is a devastating disease with severe morbidity at its advanced stages. Muscle-invasive bladder cancer (MIBC) represents an aggressive stage of bladder cancer and accounts for roughly 200,000 deaths worldwide annually^1^. The current standard of care for managing MIBC includes neoadjuvant chemotherapy followed by radical cystectomy. The most commonly used chemotherapy regimen is the gemcitabine and cisplatin (GC) combination. However, the absolute benefit of neoadjuvant chemotherapy is a modest ~5% improvement in overall survival^2–5^. To date, most studies investigating chemotherapeutic response in MIBC have conventionally focused on the direct cytotoxic effect(s) of drugs on tumor cells. In contrast, emerging evidence from other tumor types has supported the notion that select chemotherapies can recruit immune cells to target tumor cells, via inducing immunogenic cell death^6–9^. This process is facilitated by the release of immunostimulatory damage-associated molecular patterns (DAMPs; e.g., CRT, HMGB1, HSP70) by dying cancer cells, which function as immunological adjuvants for recruiting and activating dendritic cells (DCs) to subsequently promote an adaptive (T-cell-mediated) antitumoral immune response^6–10^. In contrast, as we have previously demonstrated, inhibitory DAMPs (iDAMPs; e.g., PGE_2_) functionally attenuate the immunogenicity of cell death^11,12^. Indeed, mechanistic studies have revealed that tumor-infiltrating T cells are not mere bystanders, but are important determinants that profoundly impact chemotherapeutic response^13^. However, the role(s) of immune cells (e.g., T lymphocytes and dendritic cells) in modulating chemotherapeutic efficacy in bladder cancer remain insufficiently investigated and poorly understood.

The recent FDA approval of immune-checkpoint inhibitors (ICIs) has revolutionized the treatment of advanced and metastatic MIBC; especially, for patients presenting with tumors that are pre-infiltrated with T cells (or “T-cell-inflamed” tumors)^14^. Yet, the vast majority (70–80%) of patients are nonresponsive towards ICIs; whereas the peripheral exclusion of T cells from tumor cell clusters is currently postulated as a mechanism for ICI resistance^15^. Although immune exclusion is a clinically significant phenomenon that is connected to immune-checkpoint response in bladder cancer, research progress has been significantly hampered by the lack of immune-excluded MIBC models^16^.

Aiming to improve the poor patient response towards chemotherapy or ICIs, a number of ongoing or recently completed clinical trials are combining the two as a new treatment modality (i.e., chemoimmunotherapy). Disappointingly, two highly anticipated Phase 3 clinical trials (e.g., IMvigor130 and KEYNOTE-361) evaluating the efficacy of chemoimmunotherapy in metastatic and locally advanced bladder cancer failed to improve the overall survival^17,18^. In general, there was no significant clinical benefit when comparing the overall survival or objective response of patients receiving chemoimmunotherapy to those receiving standard chemotherapy^17,18^. In retrospect, inadequate preclinical studies were performed to evaluate the immunomodulatory effects of GC chemotherapy in bladder cancer, before its hasty clinical evaluation in combination with ICIs. These recent clinical challenges underscore the necessity for a thorough preclinical study to investigate how GC chemotherapy modulates immune effector cells in bladder cancer.

Here, we report two immune-excluded murine MIBC syngeneic models that exhibit intrinsic resistance towards platinum-based chemotherapy and ICI therapy. “Inhibitory DAMP” blockade synergizes with chemotherapy in these models by inducing immunogenic cell death; thereby converting immune-excluded tumors into T-cell-inflamed. Mechanistically, iDAMP blockade skews DCs into immunogenic maturation and promotes the recruitment of antitumoral CD8+ T cells. Understanding the immunomodulatory roles of GC chemotherapy in bladder cancer is timely since these insights provide plausible explanations for the recent clinical failures of chemoimmunotherapy. Our proof-of-concept preclinical study builds upon our recent report that drug-induced immunogenic cell death is governed by an intricate balance of DAMP-to-iDAMP ratio in the tumor microenvironment^11,12^, and provides a rationale for combining “iDAMP blockade” with chemoimmunotherapy in future clinical trials.

## Results

### Generating immune-excluded murine MIBC models that are unresponsive to chemotherapy and immune-checkpoint inhibitors

**Fig. 1:**
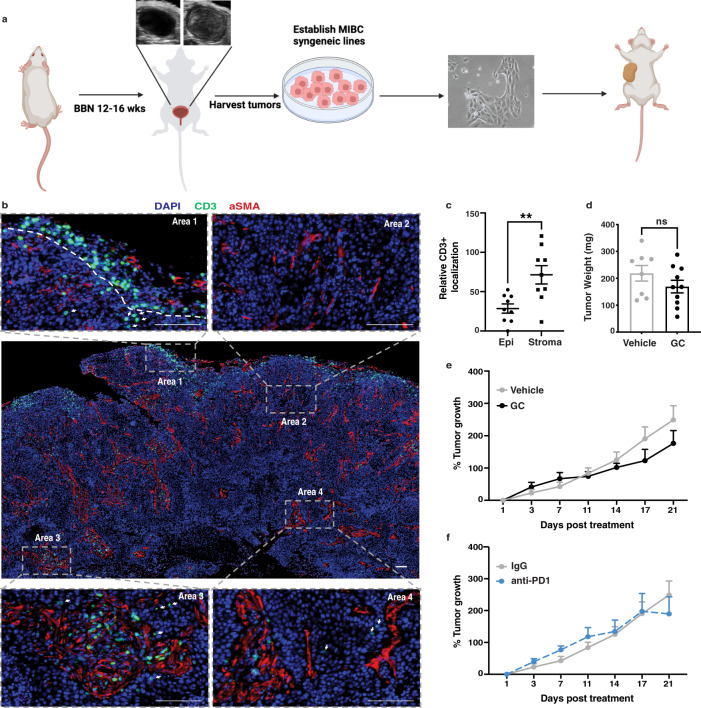
Development of a chemo- and immune-checkpoint-resistant MIBC model, with a T-cell-non-inflamed phenotype. **a** Schematic for establishment of muscle-invasive bladder cancer syngeneic lines. **b** Immunofluorescence analysis of CD3+ T-cell infiltration and aSMA expression in G69 treatment-naïve tumor tissues (Scale bar: 100 μm; Images representative of *n* = 3 independent experiments). **c** Relative localization of CD3+ T cells in treatment naïve G69 tumors. CD3+ T cells in regions of interest were quantified and normalized to average CD3+ T-cell infiltration (*n* = 9 biologically independent samples). Data are presented as mean values ± SEM. (**—Two-tailed, unpaired *t*-test *p* = 0.0045) **d, e** Weight and growth curve of G69 tumors treated with vehicle (*n* = 8 biologically independent animals) or gemcitabine (120 mg/kg) and cisplatin (6 mg/kg) (*n* = 10 biologically independent animals) for two cycles. Data are presented as mean values ± SEM. (Two-tailed, unpaired *t*-test at endpoint values). **f** Growth curve of vehicle (*n* = 8 biologically independent animals) or anti-PD1-treated (*n* = 3 biologically independent animals) (200 mg/mouse) G69 tumors monitored for 21 days. Data are presented as mean values ± SEM. (Two-tailed, unpaired *t*-test at endpoint values). Source data are provided as a Source data file. ns: not significant.

Most studies investigating therapeutic response towards ICIs in bladder cancer have focused on tumor-intrinsic mechanisms (e.g., TGF-β1, or Wnt/β-catenin)^15,19,20^. These pathways create an immunosuppressive microenvironment that precludes CD8+ T cells from the tumor parenchyma, resulting in immune-excluded (or “T-cell-non-inflamed”) bladder tumors^15,19,20^. Currently, the field faces a paucity of available murine bladder cancer models with an immune-excluded phenotype^21^, thereby, limiting the investigation of bladder cancer immunomodulation in immunocompetent hosts. We therefore generated two transplantable murine MIBC cell lines—with molecular and physiological features that are akin to the human disease—that display an immune-excluded phenotype in vivo using the following method. Mouse bladder tumors were first induced using a conventional chemical carcinogenesis model^22,23^; i.e., N-butyl-N-(4-hydroxybutyl)-nitrosamine (BBN) exposure via water intoxication as previously described^24^. Next, BBN-induced bladder tumor tissues were enzymatically processed to generate single-cell suspensions^25,26^ and were grown in vitro to establish tissue culture cell lines that allow serial transplantation in immunocompetent syngeneic hosts (Fig. 1a).

Immunofluorescence evaluation of tumor tissues from the established syngeneic MIBC lines (designated as G69 and G7 hereinafter) revealed an overall non-T-cell-inflamed phenotype (Fig. 1b, G69; Supplementary Fig. 1A, G7). The majority of CD3+ T cells (green) were excluded from the tumor cores, and instead, were contained within the tumor periphery (Fig. 1b, Area 1). Epithelial tumor regions were predominantly T cell excluded (Fig. 1b, Area 2). Minimal T cells were observed but restricted to stromal regions (αSMA+, red) intercalating between tumor clusters (Fig. 1b, Area 3 and Area 4). Quantification of CD3+ T-cell localization confirmed a stroma-restrictive pattern (Fig. 1c). In accordance with the criteria set forth by Chen & Mellman, G69 and G7 bladder tumors qualify as T-cell-non-inflamed or immune-excluded^27^.

Next, to examine how these syngeneic bladder tumors respond to standard-of-care therapies, we evaluated the efficacy of gemcitabine-cisplatin (GC) chemotherapy and ICI therapy (i.e., anti-PD1) on G69 and G7 bladder tumors in vivo. Chemotherapy failed to inhibit tumor growth (Fig. 1d, e, Supplementary Fig. 1B) and as anticipated for non-inflamed tumors, ICI therapy was also unable to suppress tumor growth (Fig. 1f, Supplementary Fig. 1C, D). Herein, we introduce and characterize two unique immune-excluded MIBC models^21^ that are unresponsive to both chemotherapeutic and ICI interventions. These represent clinically relevant murine MIBC models that recapitulate the human malady for further mechanistic investigations^21^.

### CD8+ T cells are not tumoricidal despite expressing granzyme B and IFNγ

**Fig. 2:**
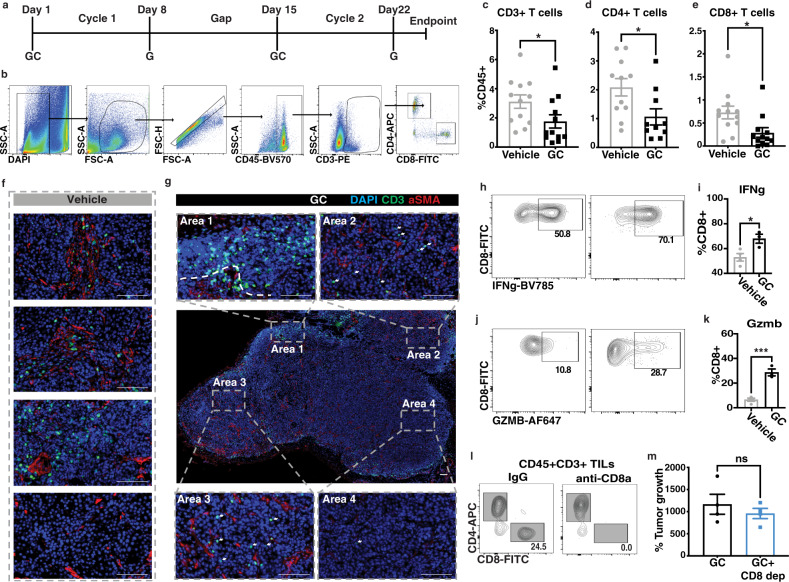
CD8+ T cells are not tumoricidal despite expressing granzyme B and IFNγ. **a** Schematic representation of chemotherapy treatment cycles. **b** Flow cytometry gating strategy for T cells from dissociated G69-tumor tissues. **c**–**e** Quantification of CD3+ T cells and their subpopulations of CD4+ and CD8+ T cells from dissociated G69-tumor tissues treated with vehicle (*n* = 12 biologically independent samples) or GC (*n* = 11 biologically independent samples) for two cycles. Data are presented as mean values ± SEM. (Two-tailed, unpaired *t*-test; *p* = 0.048, *p* = 0.023, and *p* = 0.018 respectively). **f**, **g** Immunofluorescence analysis of CD3+ T-cell infiltration in vehicle and GC-treated G69 tumors. Selected ROIs (**g**, 1–4) are representative areas that depict CD3+ T-cell localization in the tumor periphery and decreased CD3+ T-cell infiltration in the tumor (Scale bar: 100 μm; Images representative of *n* = 3 independent experiments). **h**, **i** Flow cytometry plots and quantification of IFNγ expression in CD8+ T cells from vehicle (*n* = 4 biologically independent samples) and GC-treated (*n* = 3 biologically independent samples) G69-tumor tissues. Data are presented as mean values ± SEM. (*—Two-tailed, unpaired *t*-test; *p* = 0.024). **j**, **k** Flow cytometry plots and quantification of GZMb expression in CD8+ T cells from vehicle (*n* = 4 biologically independent samples) and GC-treated (*n* = 3 biologically independent samples) G69-tumor tissues. Data are presented as mean values ± SEM. (***—Two-tailed, unpaired *t*-test; *p* = 0.0005). **l** Flow cytometry plots of CD8+ T-cell infiltration in IgG or CD8-depleted G69-tumor tissues. **m** Endpoint G69-tumor volume from mice treated with IgG or anti-CD8a specific antibody (200 mg/mouse) every other day for 2 cycles of GC (*n* = 4 biologically independent samples, respectively). Data are presented as mean values ± SEM. Source data are provided as a Source data file. ns not significant.

A recent study using bioinformatics analysis revealed that a high T-cell signature (i.e., CD3Z, CD8a, and CXCL9) in a subset of human MIBCs receiving radical cystectomy and chemotherapy positively correlated with better survival^28^. However, the immunomodulatory roles of GC chemotherapy in MIBC patients or preclinical models of MIBC are poorly characterized. To address this research gap, we sought to directly evaluate how GC chemotherapy impacts the frequency and function of T lymphocytes in our immune-excluded murine MIBC models. In brief, mice bearing G69 and G7 tumors were treated with two cycles of GC chemotherapy with a rest period in between to recapitulate the clinical management of MIBC^25^ (Fig. 2a). Forty-eight hours after the completion of the second GC cycle, tumor tissues were harvested and analyzed for the presence of tumor-infiltrating lymphocytes by flow cytometry (Fig. 2b). Treatment with GC chemotherapy significantly decreased overall tumor-infiltrating CD3+ T cells, as well as CD4+ and CD8+ T-cell subsets when compared to vehicle-treated tumors (Fig. 2c–e; *P < 0.05, **P < 0.001). While GC chemotherapy decreased the overall frequency of tumor-infiltrating T cells, immunofluorescence analysis revealed further spatial information which showed an increase in “immune desert” areas toward the core of the chemotherapy-treated tumors (Fig. 2g, Area 4). The majority of CD3+ T cells were constrained at the periphery (Fig. 2g, Area 1), while GC chemotherapy seemed to slightly increase T-cell infiltration into the epithelial region in certain areas (Fig. 2g, Area 2 and Area 3).

Given the importance of CD8+ T cells as effectors for eliminating cancer cells, we evaluated the co-expression of IFNγ and GZMb in CD8+ TILs—two commonly used markers to indicate their activation status and antitumoral activities^29^. CD8+ TILs from GC-treated tumors exhibited a significant increase in the expression of IFNγ and GZMb when compared to vehicle treatment alone (Fig. 2h, i and j, k, respectively; *P < 0.05, ***P < 0.005). Despite the expression of these active marks, functional evaluation of CD8+ T cells via antibody depletion in vivo (Fig. 2l) failed to exhibit changes in tumor growth when compared to IgG-treated controls (Fig. 2m), indicating they were nontumoricidal functionally. Taken together, these results demonstrate that GC chemotherapy fails to mount an effective antitumoral T-cell response in immune-excluded MIBC.

### Gemcitabine-cisplatin chemotherapy attenuates the maturation of CD11c + MHCII + dendritic cells

**Fig. 3:**
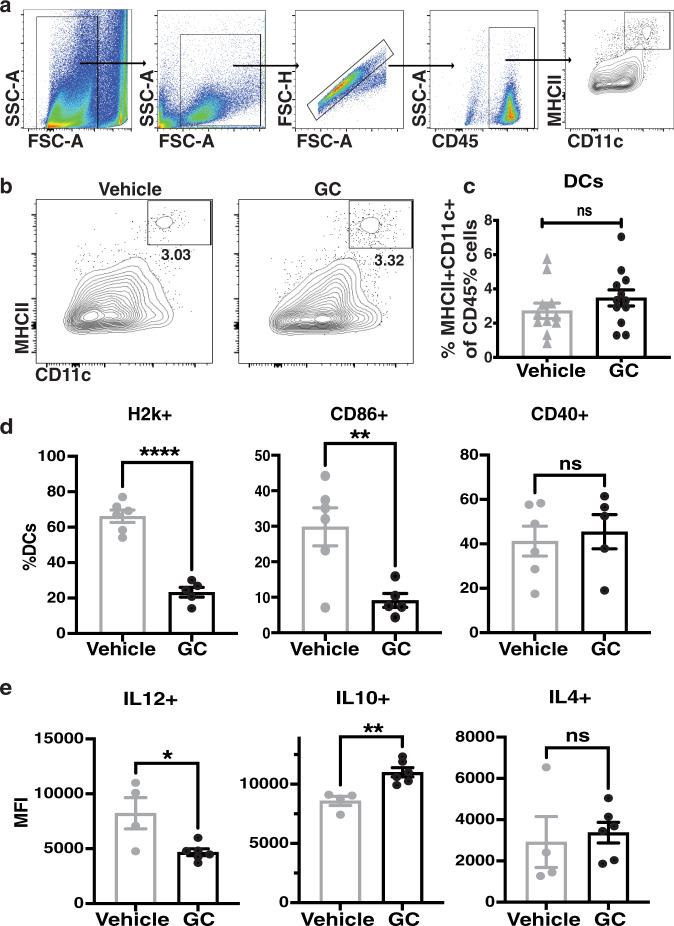
Gemcitabine-cisplatin chemotherapy attenuates the maturation of immunogenic CD11c + MHCII + dendritic cells. **a** Flow cytometric gating strategy for dendritic cells from G69-tumor tissues. **b, c** Flow cytometry plots and corresponding quantification of CD11c + MHCII + dendritic cells (DC) from vehicle and GC-treated G69-tumor tissues (*n* = 11 and *n* = 12 biologically independent samples respectively). Data are presented as mean values ± SEM. **d** Relative quantification (%CD11c + MHC2+) of H2-K, CD86, and CD40 expression in tumor-infiltrating DCs from vehicle and GC-treated G69-tumor tissues (*n* = 6 and *n* = 5 biologically independent samples, respectively). Data are presented as mean values ± SEM. (Two-tailed, unpaired *t*-test. ****p < 0.0001, **p = 0.008). **e** Mean fluorescence intensity (MFI) of IL12, IL10, and IL4 expression in CD11c + MHC2 + DCs from vehicle or GC-treated G69-tumor tissues (*n* = 4 and *n* = 6 biologically independent samples, respectively). Data are presented as mean values ± SEM. (Two-tailed, unpaired *t*-test. *p = 0.018, **p = 0.003). Source data are provided as a Source data file. ns not significant.

To understand why these CD8+ T cells were nontumoricidal, we explored the impact of GC chemotherapy on modulating dendritic cell maturation within G69 bladder tumors. Since proper priming of CD8+ T cells by mature dendritic cells (DCs) is essential for the generation of antigen-specific cytotoxic T lymphocytes with antitumor activities^30,31^, we sought to explore this interaction. In brief, immunogenic DCs prime antitumoral CD8+ T cells through providing T-cell receptor activating signal (signal 1; MHCI), co-stimulation (signal 2; CD86, CD40) and paracrine stimuli (signal 3; cytokines); important for the proper activation, propagation, and polarization of naive CD8+ T cells into effector cells^32^. We, therefore, analyzed the infiltration and maturation status of DCs in vehicle- and GC-treated tumors using flow cytometry (Fig. 3a). Between the treatment groups, DCs were detected within all tumors and no difference in frequency was observed (Fig. 3b, c). However, further evaluation revealed that DCs in GC-treated tumors exhibited a significantly reduced expression of H2-k (i.e. MHCI; Signal 1) and CD86 (Signal 2) (Fig. 3d), indicative of diminished DC activation. In addition, the expression of a key Th1-polarizing cytokine, IL12, was significantly decreased in DCs from GC-treated tumors as well (Fig. 3e, left panel). Conversely, the expression of a pro-tumoral immunosuppressive, class 2 cytokine, IL10^33^, was significantly increased (Fig. 3e, middle panel), while no difference was observed in the expression of the Th2-polarizing cytokine, IL4 (Fig. 3e, right panel). These results revealed that dendritic cells in GC-treated tumors expressed markers indicative of a diminished maturation and Th1-promoting cytokine^34^.

### Gemcitabine-cisplatin chemotherapy promotes inhibitory DAMP release to attenuate dendritic cell maturation

To assess how GC chemotherapy diminished DC maturation, we first investigated whether GC chemotherapy impacts the release of DAMPs and inhibitory DAMP (iDAMP). The success of drug-induced immunogenecity is contingent on the cell-surface expression or extracellular release of immunological adjuvants (i.e., DAMPs) by dying cancer cells to facilitate the maturation of DCs, leading to the priming and recruitment of CD8+ T cells^8,35^. Most recently, we highlighted an intricate balance between immunostimulatory and inhibitory DAMP release that converges as “signal 0” to dictate the maturation of dendritic cells^11^. Considering GC chemotherapy resulted in a diminished infiltration of CD8+ T cells (Fig. 2a–j) and attenuated DC maturation (Fig. 3a–e) in vivo, we examined how GC chemotherapy impacted the release of bona fide immunostimulatory DAMPs (i.e., CRT, HSP70, and HMGB1) and the inhibitory DAMP (PGE_2_) in vitro and in vivo. Cultured G69 and G7 cells treated with IC50 concentrations of GC chemotherapy displayed increased cell-surface expression of CRT and HSP70 compared to vehicle-treated cells (Supplementary Fig. 2A); and accordingly, increased HMGB1 extracellular release into the cultured media (Supplementary Fig. 2B). Furthermore, both G69 and G7 syngeneic tumors treated with GC chemotherapy revealed an increased serum expression of HMGB1 (Supplementary Fig. 2C, ELISA), when compared with vehicle-treated controls. Taken together, these results confirmed GC chemotherapy induces the release of immunostimulatory DAMPs in vitro and in vivo.

**Fig. 4:**
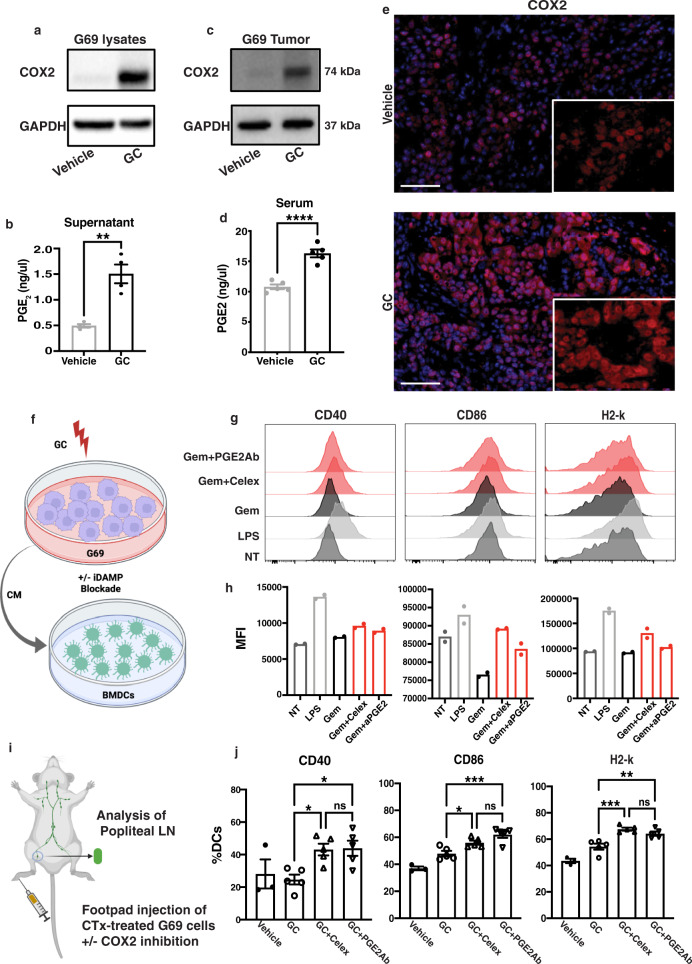
Gemcitabine-cisplatin chemotherapy promotes an overwhelmingly high release of inhibitory DAMP to attenuate DC maturation. **a** Western blot analysis of COX-2 expression in G69 cell lysates treated with either vehicle or GC for 48 h. GAPDH expression was used as loading control (*n* = 3 biologically independent experiments). **b** ELISA for PGE_2_ release in the culture medium of G69 cells treated with either vehicle control or GC for 48 h (*n* = 4 independent biological samples). Data are presented as mean values ± SEM. (Two-tailed, unpaired *t*-test **p = 0.0016). **c** COX-2 expression in G69-tumor tissue lysates treated with either vehicle or GC for two chemotherapy cycles. GAPDH expression was used as a loading control (*n* = 3 biologically independent experiments). **d** ELISA for PGE2 release in the serum of G69-tumor-bearing mice treated with either vehicle or GC for 2 cycles (*n* = 5 and *n* = 4 biologically independent samples for vehicle and GC, respectively). Data are presented as mean values ± SEM. (Two-tailed, unpaired *t*-test ****p < 0.0001). **e** Immunofluorescence analysis of COX-2 expression in G69-tumor tissues treated with either vehicle or GC for two chemotherapy cycles (Scale bar: 100 μm; Images representative of *n* = 3 independent experiments). **f** Schematic representation of BMDC treatment with conditioned media in the presence or absence of iDAMP blockade. **g** Flow cytometric analysis of CD40, CD86, and MHCI expression in BMDCs treated with G69-conditioned media with or without iDAMP blockade. **h** Geometric mean fluorescence intensity (gMFI) of CD40, CD86, and MHCI molecule expression in BMDCs upon treatment with condition media as in **g** (*n* = 2 biologically independent samples). Data are presented as mean values. **i** Schematic representation of footpad vaccination assay. **j** Flow cytometric analysis of CD40, CD86 and MHCI expression in vaccination-draining lymph node CD11c + MHC2 + DCs upon injection of G69 cells treated with GC in the presence or absence of iDAMP blockade (*n* = 5 biologically independent samples). Non-vaccination-draining lymph node DCs were used as control (*n* = 3 biologically independent samples). Data are presented as mean values ± SEM. (One-way ANOVA-Tukey’s multiple comparisons test. CD40: GC vs GC+ Celex, *p* = 0.045; GC vs GC+ aPGE2, *p* = 0.036; CD86: GC vs GC+ Celex, *p* = 0.046; GC vs GC+ aPGE2, *p* = 0.0007; H2-K: GC vs GC+ Celex, *p* = 0.0004; GC vs GC+ aPGE2, *p* = 0.005). Source data are provided as a Source data file. ns not significant.

Coinciding immunostimulatory DAMP release, GC chemotherapy-treated G69 cancer cells displayed increased COX-2 protein expression (i.e., upstream prostaglandin metabolizing enzyme, Fig. 4a), resulting in the elevated release of the inhibitory DAMP (i.e., PGE_2_) into the cultured medium, when compared to vehicle-treated cells (Fig. 4b). Gemcitabine-cisplatin chemotherapy-treated tumors in vivo also exhibited increased protein expression of COX-2 (Fig. 4c), and upregulated release of PGE_2_ into serum when compared to vehicle-treated mice (Fig. 4d). Furthermore, COX-2 upregulation was validated by immunofluorescence analysis comparing vehicle- and GC-treated tumor tissues (Fig. 4e). Taken together, these results supported our previous finding that extracellular release of the inhibitory DAMP (or PGE_2_) counteracted the adjuvanticity of immunostimulatory DAMPs in precluding DC maturation^11^.

Next, we assessed the immunomodulatory effects of DAMPs and iDAMP on DC maturation by two independent methods. First, we collected conditioned media from GC chemotherapy-treated G69 cells (Fig. 4f). We then generated bone-marrow-derived DCs (BMDCs) by culturing bone marrow cells following a standard protocol using GM-CSF, as previously described^36^ (Fig. 4g). These BMDCs were subsequently cultured in conditioned media from G69-tumor cells pre-treated with GC chemotherapy in the presence or absence of iDAMP blockade (Fig. 4f, g). The inhibitory DAMP blockade was achieved by two methods: (i) treatment of G69 cells with GC chemotherapy in the presence of celecoxib—a COX-2 specific inhibitor, and (ii) a PGE_2_ neutralizing antibody to sequester PGE_2_ in the culture media. BMDCs were treated with these conditioned media for 24 h and harvested for flow cytometric analyses of DC maturation markers (Fig. 4g). Inhibitory DAMP blockade by both approaches consistently induced increased expression of maturation markers (i.e., CD40, CD86, H2-k) in BMDCs (Fig. 4g, red histograms), which was compared with LPS-stimulated BMDCs (as positive control) and untreated tumor cells (as NT negative control) (Fig. 4g, light gray and dark gray histograms, respectively). As anticipated, BMDCs cultured in conditioned media without iDAMP blockade failed to upregulate DC maturation markers (Fig. 4g, dark gray histograms). The mean fluorescent intensities (MFIs) for each of these conditions are also presented in Fig. 4h as bar graphs. As a complementary approach, we adapted and performed a classical in vivo vaccination assay^11,37^. Gemcitabine-Cisplatin chemotherapy-treated G69 cancer cells (IC50 dose to induce cell death) in the presence or absence of iDAMP blockade were injected into the mouse footpad as “vaccines”. The maturation status of dendritic cells from vaccine-treated draining lymph nodes was evaluated 3 days post-injection (Fig. 4i, j). Flow cytometric analysis of popliteal lymph node revealed iDAMP blockade increased the expression of DC maturation markers (i.e., CD40, CD86, and H2-k), when compared to GC chemotherapy-treated group control (Fig. 4j). Taken together, these results demonstrated that functional inhibition of the COX-2/PGE_2_ axis promotes DC maturation in vitro and in vivo.

### iDAMP blockade inhibits tumor growth by promoting pro-inflammatory DC maturation

**Fig. 5:**
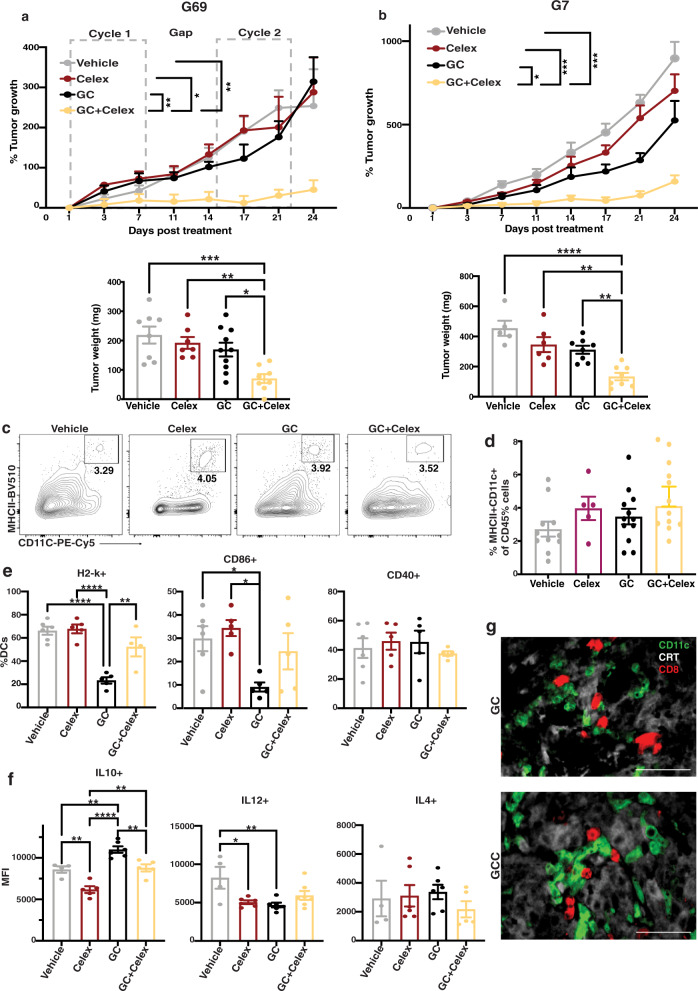
iDAMP blockade inhibits tumor growth by promoting pro-inflammatory DC maturation. **a** Percent growth curves (top) and tumor weight (bottom) of G69 tumors from vehicle (*n* = 7 biologically independent animals), Celecoxib (*n* = 7 biologically independent animals), GC (*n* = 8 biologically independent animals), and GC+ Celecoxib-treated mice (*n* = 7 biologically independent animals) upon two cycles of chemotherapy. Percent tumor growth was obtained by normalizing to starting tumor volumes. Doted gray rectangles denote the starting point and the end point of each GC cycle. Celecoxib was injected ip 48 h before GC treatment initiation and continued daily to endpoint. Data are presented as mean values ± SEM. (Top: One-way ANOVA-Tukey’s multiple comparisons test. GC+ Celex vs Vehicle *p* = 0.006; vs Celex *p* = 0.029; vs GC *p* = 0.001; Bottom: One-way ANOVA-Tukey’s multiple comparisons test. GC+ Celex vs Vehicle *p* = 0.0006; vs Celex *p* = 0.007; vs GC *p* = 0.019). **b** Percent growth curves (top) and tumor weight (bottom) of G7 tumors from vehicle (*n* = 5 biologically independent animals), Celecoxib (*n* = 6 biologically independent animals), GC (*n* = 8 biologically independent animals), and GC+ celecoxib-treated mice (*n* = 8 biologically independent animals) upon two cycles of chemotherapy. Data are presented as mean values ± SEM. (One-way ANOVA-Tukey’s multiple comparisons test. GC+ Celex vs Vehicle; vs Celex *p* = 0.0002; vs Celex *p* = 0.0003; vs GC *p* = 0.028; Bottom: One-way ANOVA-Tukey’s multiple comparisons test. GC+ Celex vs Vehicle *p* < 0.0001; vs Celex *p* = 0.002; vs GC *p* = 0.004). **c**, **d** Flow cytometry plots and corresponding quantification of CD11c + MHCII + dendritic cells (DC) from vehicle (*n* = 11 biologically independent samples), Celecoxib (*n* = 5 biologically independent samples), GC (*n* = 12 biologically independent samples), and GC+ Celecoxib-treated (*n* = 12 biologically independent samples) G69-tumor tissues. Data are presented as mean values ± SEM. **e** Relative quantification (%CD11c + MHC2+) of H2-K, CD86, and CD40 expression in tumor-infiltrating DCs from vehicle (*n* = 6 biologically independent samples), celecoxib (*n* = 5 biologically independent samples), GC (*n* = 5 biologically independent samples), and GC+ Celecoxib-treated (*n* = 4 biologically independent samples) G69-tumor tissues. Data are presented as mean values ± SEM. (One-way ANOVA-Tukey’s multiple comparisons test. H2-k: Vehicle vs GC *p* < 0.0001, Celex vs GC *p* = <0.0001, GC vs GC+ Celex *p* = 0.004; CD86:Vehicle vs GC *p* = 0.047, Celex vs GC *p* = 0.038). **f** Mean fluorescence intensity (MFI) of IL12, IL10, and IL4 expression in CD11c + MHC2 + DCs from vehicle (*n* = 4 biologically independent samples), celecoxib (*n* = 5 biologically independent samples), GC (*n* = 6 biologically independent samples), and GC+ Celecoxib-treated (*n* = 5 biologically independent samples) G69-tumor tissues. Data are presented as mean values ± SEM. (One-way ANOVA-Tukey’s multiple comparisons test. IL10: Vehicle vs Celex *p* = 0.004, vs GC *p* = 0.006, Celex vs GC *p* = <0.0001, vs GC+ Celex *p* = 0.002, GC vs GC+ Celex *p* = 0.005; IL12:Vehicle vs Celex *p* = 0.02; vs GC *p* = 0.009). **g** Representative images of immunofluorescence analysis of CD8+ T cells (red) interaction with CD11c + DCs (green) and CRT + G69 cells (white) upon treatment with either GC or GC+ Celecoxib for 2 cycles (Scale bar: 100 μm; Images representative of *n* = 3 independent experiments). Source data are provided as a Source data file.

To determine whether iDAMP blockade in preclinical models recapitulate its effects on modulating DC maturation, we treated G69 and G7 tumor-bearing mice with two cycles of GC chemotherapy in the presence or absence of celecoxib. In vivo celecoxib treatment was initiated 2 days prior to the first chemotherapy treatment and was administered daily for the duration of the experiment. The combination of GC chemotherapy with celecoxib (GC+ Celex) resulted in a significantly reduced tumor growth (and tumor weight at endpoint), when compared to GC chemotherapy treatment alone (Fig. 5a, b).

To evaluate the mechanisms underlying these significant differences in tumor volume, we examined whether celecoxib or iDAMP blockade promotes DC maturation in vivo. Co-treatment with celecoxib did not alter DC infiltration into tumors (Fig. 5c, d), as assessed by flow cytometry. Remarkedly, DCs from GC+ celecoxib-treated tumors exhibited significantly increased H2-k expression, when compared to DCs from GC-treated tumors (Fig. 5e, left panel). CD86 expression also exhibited a similar trend and was comparable to the vehicle control group; however, some variations existed between samples (Fig. 5e, middle panel). No difference was observed in CD40 expression (Fig. 5e, right panel). Importantly, PGE_2_ blockade in vivo normalized IL12 expression to vehicle levels, while decreasing IL10 expression in tumor DCs (Fig. 5f left and middle panel, respectively). No difference was observed in the expression of IL4 (Fig. 5f, right panel). Evidence of DC interactions with CD8+ T cells was observed in tissues from both GC and GC+ Celex-treated tumors as expected (Fig. 5g), indicative of CD8+ T-cell priming at the tumor site.

### iDAMP blockade-mediated tumor suppression is dependent on functional CD8+ T cells

**Fig. 6:**
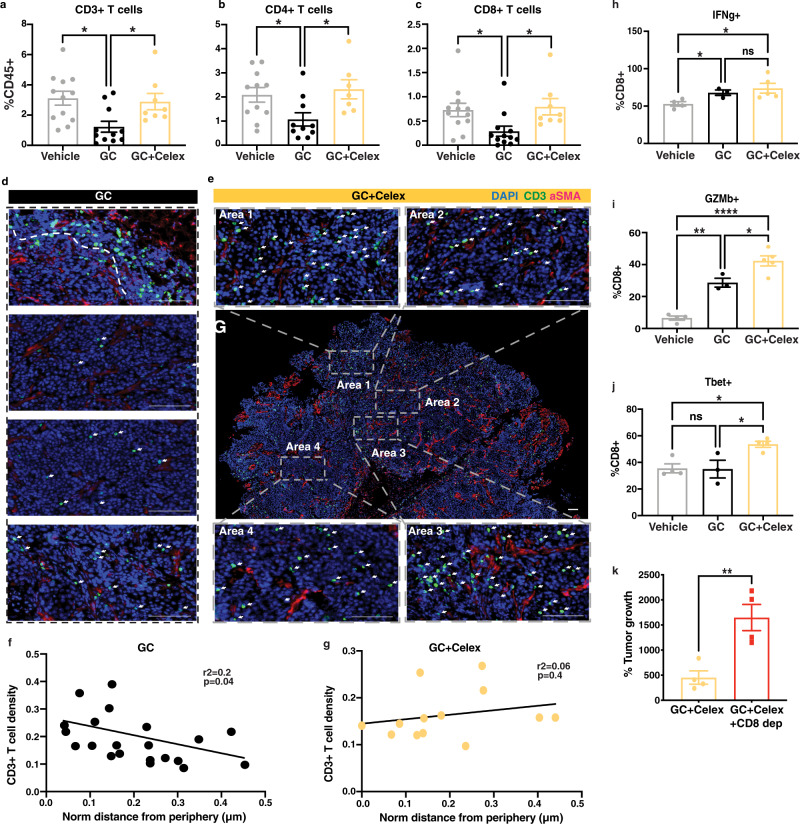
iDAMP blockade-mediated tumor suppression is dependent on functional CD8+ T cells. **a**–**c** Quantification of CD3+ T cells and their subpopulations of CD4+ and CD8+ T cells from dissociated G69-tumor tissues treated with vehicle (*n* = 12 biologically independent samples), GC (*n* = 11 biologically independent samples), or GC+ celecoxib (*n* = 8 biologically independent samples) for two cycles. Data are presented as mean values ± SEM. (One-way ANOVA-Tukey’s multiple comparisons test. CD3: GC vs Vehicle *p* = 0.01, vs GC+ Celex *p* = 0.049; CD4: GC vs Vehicle *p* = 0.047, vs GC+ Celex *p* = 0.043; CD8: GC vs Vehicle *p* = 0.048, vs GC+ Celex *p* = 0.048). **d**, **e** Immunofluorescence analysis of CD3+ T-cell infiltration in vehicle, GC, and GC+ Celecoxib-treated G69 tumors. Selected ROIs (1–4) are representative areas that depict CD3+ T-cell infiltration in the tumor core and decreased presence of CD3+ T cells in the tumor periphery (Scale bar: 100 μm; Images representative of *n* = 3 independent experiments). **f**, **g** Relative CD3+ T cells density as a function of the distance from tumor periphery for GC (*n* = 5 biologically independent samples) and GC+ Celecoxib-treated (*n* = 3 biologically independent samples) G69-tumor section. CD3+ T-cell density was calculated as described in materials and methods. Graphs depict CD3+ T cells density versus distance from tumor periphery for 3–5 ROIs from each sample. **h**–**j** Flow cytometric quantification of IFNg, GZMb, and Tbet expression in CD8+ T cells from vehicle (*n* = 4 biologically independent samples), GC (*n* = 3 biologically independent samples), and GC+ Celecoxib-treated (*n* = 5 biologically independent samples) G69-tumor tissues. Data are presented as mean values ± SEM. (One-way ANOVA-Tukey’s multiple comparisons test. IFNg: GC vs Vehicle *p* = 0.024, vs GC+ Celex *p* = 0.042; GZMb: Vehicle vs GC *p* = 0.001, vs GC+ Celex *p* < 0.0001, GC vs GC+ Celex *p* = 0.019; Tbet: GC+ Celex vs Vehicle *p* = 0.02, vs GC *p* = 0.03). **k** Endpoint G69-tumor volume from mice treated with IgG or anti-CD8a specific antibody (200 mg/mouse) every other day for 2 cycles of GC+ Celecoxib (*n* = 4 biologically independent animals). Data are presented as mean values ± SEM. (Two-tailed, unpaired *t*-test *p* = 0.006). Source data are provided as a Source data file. ns not significant.

To further evaluate how GC+ celecoxib mediated DC maturation (Fig. 5e, f) might impact T cells in vivo, we investigated whether iDAMP/PGE_2_ blockade affects infiltration CD3+ T cells into tumors or polarization of CD8+ cytotoxic T cells into (Tc)-1 or 2 phenotypes. To evaluate this, we analyzed tumor tissues treated with GC+ celecoxib and other control groups by flow cytometry. Indeed, iDAMP blockade resulted in a significantly increased CD3+ T-cell infiltration as well as CD4+ and CD8+ T-cell subsets when compared with the GC-treated bladder tumors (Fig. 6a–c). Further immunofluorescence analysis revealed additional spatial redistribution of CD3+ TILs in mice with iDAMP blockade (Fig. 6d, e, GC+ Celex), which are not reflected by flow cytometry analysis alone (Fig. 6a). Celecoxib enhanced the infiltration of T cells into the tumor core (Fig. 6e, Area 1–4) with a simultaneous decrease of CD3+ TILs in the tumor periphery when compared to GC-treated tumor tissues (Fig. 6d, e, quantified in 6F-G). Furthermore, we also evaluated the activation status of CD8+ T cells upon iDAMP blockade (i.e., GC+ Celex group). Flow cytometric analysis of CD8+ TILs revealed an increased expression of effector molecules such as IFNγ and GZMb upon iDAMP blockade (Fig. 6h, i and Supplementary Fig. 3A, B), as well as an increase in Tbet expression (Fig. 6j and Supplementary Fig. 3C) compared to their GC-treated counterparts (i.e., GC group). Importantly, these reinvigorated CD8+ TILs were functionally tumoricidal, and indeed responsible for the improved efficacy of GC chemotherapy, since their functional depletion (via antibody) nullified the effects of iDAMP blockade (Fig. 6k). Collectively, the above results illustrate that iDAMP blockade reprograms the tumor microenvironment by facilitating a CD8+ Tc1-mediated antitumor immune response and tissue infiltration.

### iDAMP blockade synergizes with chemotherapy to enhance immune-checkpoint response

While recent FDA approval of ICIs revolutionized the treatment of advanced and metastatic MIBCs, only a subset of MIBC patients is considered responders to ICIs (~15–30%)^38^. To address this unmet clinical need, several clinical trials are underway or have concluded in evaluating the combination of ICIs with GC chemotherapy. Disappointingly, two Phase 3 clinical trials combining ICIs with GC chemotherapy [IMvigor 310 and KEYNOTE-361] recently failed to meet their primary endpoint on improving the overall survival or objective response over monotherapies^17,18^, posing a new clinical challenge. Based on our preclinical findings that GC chemotherapy-induced a significant reduction of tumor-infiltrating T cells (Fig. 2a–g) and rendering them nontumoricidal (Fig. 2m), it might not be unreasonable that these Phase 3 clinical trials have resulted in disappointing efficacies.

**Fig. 7:**
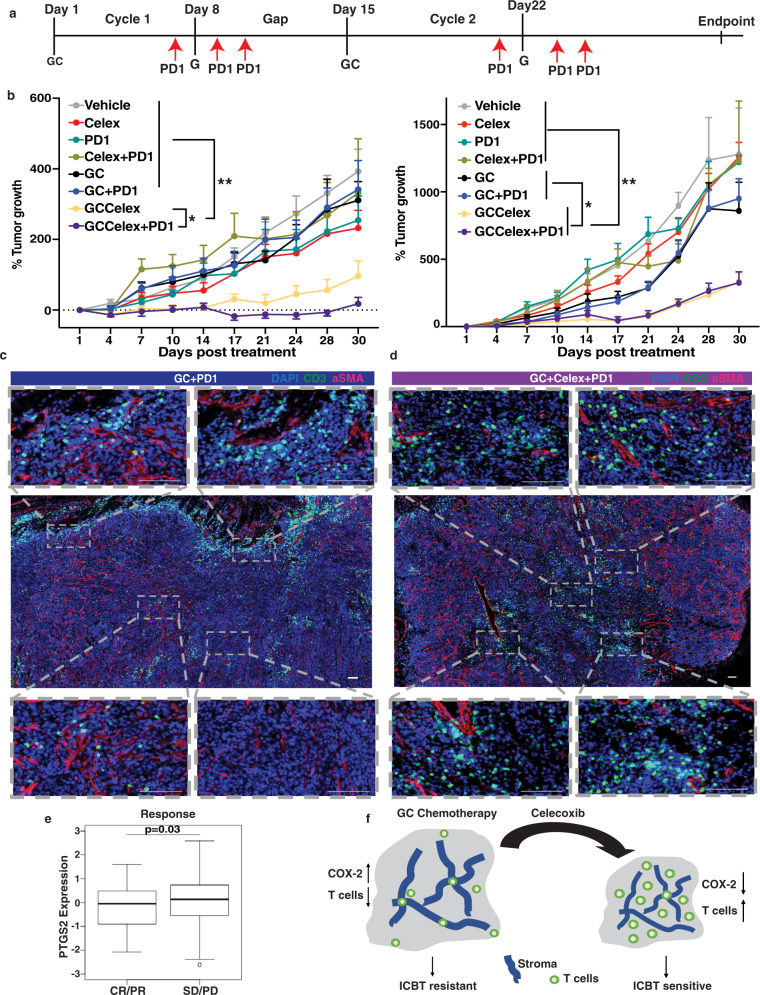
iDAMP blockade synergizes with chemotherapy to enhance immune-checkpoint response. **a** Schematic representation of ICBT incorporation into the GC treatment cycles. Anti-PD1 (clone: 29F-1A12) was injected ip on days 7, 9, and 11 of each GC cycle. **b** G69 (left panel) and G7 (right panel) tumor growth curves of vehicle (*n* = 8 biologically independent animals), Celecoxib (*n* = 7 biologically independent animals), anti-PD1 (*n* = 7 biologically independent animals), Celecoxib+anti-PD1 (*n* = 6 biologically independent animals), GC (*n* = 8 biologically independent animals) and GC+ anti-PD1 (*n* = 6 biologically independent animals), GC+ Celecoxib (*n* = 7 biologically independent animals), and GC+ Celecoxib + anti-PD1-treated mice (*n* = 8 biologically independent animals) for two cycles of chemotherapy. Data are presented as mean values ± SEM. (One-way ANOVA-Tukey’s multiple comparisons test. Left: *p = 0.045, **p = 0.005; Right: *p = 0.013, **p = 0.007). **c**, **d** Immunofluorescence analysis of CD3+ T-cell infiltration in GC+ PD1, and GC+ Celecoxib+PD1-treated G69 tumors. Selected ROIs (1–4) are representative areas that depict CD3+ T-cell infiltration in the tumor core and the tumor periphery (Scale bar: 100 μm; Images representative of *n* = 3 independent experiments). **e** PTGS2 (COX-2 gene) expression in complete/partial responders (CR/PR, *n* = 68) and stable/progressive disease (SD/PD, *n* = 230) MIBC patients from chemotherapy-ineligible or refractory MIBC patient cohort (Imvigor210) treated with atezolizumab (anti-PD-L1). (Wilcoxon paired two-sided rank-sum test *p*  = 0.03). Boxplots are drawn as the inter-quartile range (IQR) with a line indicating the median, and outliers defined as points that fall outside of the range demarcated by 1.5×IQR. **f** Schematic representation of COX-2 blockade as a valid therapeutic target to improve chemoimmunotherapy response for MIBC patients with immune-privileged tumors. Source data are provided as a Source data file.

Given the impact of iDAMP blockade in improving the infiltration of tumoricidal CD8+ T cells into the tumor core when combined with GC chemotherapy (Fig. 6e), we hypothesize that iDAMP blockade likely poses a superior option to combine with chemoimmunotherapy (Fig. 7a). We treated G69 and G7 tumor-bearing mice with different drug combinations (Fig. 7a), following the same treatment scheme in Fig. 2a with the addition of anti-PD1 drug on day 7, 9, and 11 of cycle 1 and days 21, 23, 25 of cycle 2 (Fig. 7a). The anti-PD1 treatment scheme was conceived after considering the time needed for the immune system to mount an initial response to tumor neo-antigens (~8 days)^39^. As expected, a combination of GC chemotherapy with anti-PD1 exhibited no effect on tumor growth when compared to celecoxib- or GC-treated tumors (Fig. 7b), recapitulating the disappointing results from recent Phase 3 clinical trials^17^. Remarkably, iDAMP blockade via celecoxib demonstrated prolonged suppression of tumor growth when combined with GC chemotherapy and anti-PD1 (Fig. 7b, purple line; left panel), significantly superior to GC chemotherapy and celecoxib combination (Fig. 7b, blue line; left panel) in the G69 model. For the G7 syngeneic model, it is intriguing that the GC chemotherapy and celecoxib combination is as efficient as the GC chemotherapy plus anti-PD1 and celecoxib (Fig. 7b, right panel). It is also important to highlight that GC+ Celex (Fig. 7b, blue line) is significantly superior to GC plus anti-PD1 (Fig. 7b, yellow line), which essentially has no effects over GC chemotherapy alone (Fig. 7b, black line) for both G69 and G7 cancer models. Further immunofluorescence analyses of the treated tumors revealed that CD3+ T cells are primarily excluded along the tumor periphery (Fig. 7c, Area 1 and 2) or within stromal regions (Fig. 7c, Area 3 and 4) in the GC+ PD1 group. Remarkedly, iDAMP blockade induced T-cell infiltration into the epithelial tumor cell regions (Fig. 7d, Area 1–4; GC+ Celex+PD1 group), supportive of the remarkable therapeutic response in Fig. 7b.

### High PTGS2 expression in pretreatment tissues correlates with poor ICI response in MIBCs

We have previously shown that *PTGS2* (gene that encodes COX-2) expression is significantly upregulated in matching pre- versus post-chemotherapy-treated MIBC patients that are chemotherapy nonresponders^25,40^. To evaluate whether *PTGS2* expression exhibits an association with ICI response in MIBC patients, we analyzed its expression in the IMvigor210 study where patients received ICI after they failed platinum-based chemotherapy^41^. Intriguingly, *PTGS2* expression in pretreatment tissues significantly correlated with response to ICI therapy. Bladder cancer patients with stable or progressive disease (SD/PD, *n* = 230) exhibited a significantly higher *PTGS2* gene expression when compared to patients demonstrating complete or partial response (CR/PR, *n* = 68) (*p* = 0.03, Fig. 7e).

Collectively, in conjunction with the preclinical studies in Fig. 7a–d, the clinical correlative studies in Fig. 7e confirmed *PTGS2/*COX-2 as a clinically relevant target, and pose “iDAMP blockade” as a rational combination with GC chemotherapy and ICI to be evaluated in future clinical trials (Fig. 7f).

## Discussion

The advent of immune-checkpoint inhibitors (ICIs) has revolutionized the management of advanced and metastatic bladder cancer patients, which has remained stagnant for decades^38^. Despite the impressive survival benefit and durable response in a subset of patients (15–30%) characterized as responders to ICIs, they are usually limited to those with “inflamed tumors” (i.e., high T-cell infiltration in the tumor parenchyma)^38,42^. Recent efforts to increase the proportion of bladder cancer patients that can benefit from ICIs have primarily focused on combining ICIs with cisplatin-based chemotherapies. This combinatorial treatment modality (i.e., chemoimmunotherapy) has been tested in various cancer types––such as lung (IMpower130, Keynote 189), breast (IMpassion130), as well as head and neck cancers (Keynote 048)—where a significant improvement in overall patient survival was achieved^43–46^. However, at least one Phase 2 (NCT01524991) and two Phase 3 clinical trials in metastatic or locally advanced bladder cancer (IMvigor130 and KEYNOTE-361) have failed to demonstrate significant clinical benefit, when comparing the overall survival of bladder cancer patients who received chemoimmunotherapy to those who received standard chemotherapy^17,18,47^. These adverse outcomes suggest that additional, unexplored mechanisms such as the understudied bladder tumor microenvironment may influence the therapeutic efficacy of chemoimmunotherapy in this cancer type^48^.

In hindsight, insufficient preclinical studies were performed to evaluate the immunomodulatory roles of GC chemotherapy in bladder cancer, before its combination with ICIs in large-scale human clinical trials. One major technical challenge that has hampered such research in the field is the paucity of preclinical MIBC models that recapitulate the intratumoral immune profiles of human MIBC. For instance, in a high-profile study of bladder cancer, immune exclusion was postulated to be a clinically significant phenomenon that correlated with immune-checkpoint resistance^19^; yet, the authors had to defer to a murine breast cancer model for mechanistic studies. Similarly, in another elegant study that evaluated the immunogenic effects of mitomycin C chemotherapy in the non-muscle-invasive bladder cancer setting, the authors deferred to a murine colon cancer model for mechanistic evaluation^49^. These are important studies investigating clinically relevant phenomena in human MIBCs. Therefore, this lack of relevant preclinical MIBC models underscores the significance of our current study in the development and characterization of autochthonous, immune-excluded MIBC mouse models (i.e., G69 and G7) for mechanistic investigations.

Using these immune-excluded MIBC models (i.e., G69 and G7), we found that standard GC chemotherapy significantly suppressed the frequency of intratumoral T-cell infiltration and increased the overall immune desert areas towards the center or core of the tumors. Although these tumor-infiltrating CD8+ T cells expressed classical markers indicative of their activation, functional evaluation via antibody depletion unexpectedly revealed they were nontumoricidal. These results suggest several plausible explanations. First, these CD8+ T cells were indeed activated but excluded to the tumor periphery; and therefore, unable to eliminate cancer cells due to their lack of tissue penetration into the tumor core. Secondly, since dendritic cells (DCs) are important partners for proper priming and activation of CD8+ T cells^50^, these tumor-infiltrating CD8+ T cells might not be properly primed by DCs. Indeed, the mechanistic investigation revealed that chemotherapy exerted negative effects to skew DC away from maturation and immunogenic polarization, which was also accompanied by an increased expression of the immunosuppressive cytokine, IL10, and a decreased expression of the Th1-polarizing cytokine, IL12. The upregulation of IL10 and downregulation of IL12 has been linked to anti-inflammatory responses that support the development of a pro-tumoral microenvironment^33^. Further mechanistic dissection also revealed an overwhelming increase of extracellular PGE_2_ release from chemotherapy-treated cancer cells, a mechanism to hinder immunogenic DC maturation. These preclinical findings in vivo substantiated our recent findings posing extracellular PGE_2_ as an inhibitory DAMP to counterpoise immunostimulatory DAMPs, negating their downstream signaling on dendritic cells to activate immunogenic cell death^11^. Using both in vitro and in vivo models, we demonstrated that pharmaceutical blockade of PGE_2_ synthesis into the tumor microenvironment enhanced immunogenic DC maturation; thereby, reverting the tolerogenic status of DCs within the GC chemotherapy group. These effects of the COX-2/PGE_2_ axis on dendritic cells corroborate with a previous study in a different context and cancer type^51^. In brief, tumor-derived PGE_2_ has been reported to inhibit CD103+ DC recruitment into the tumor microenvironment in the treatment naïve setting by promoting melanoma tumor growth through immune evasion^51^. In other MIBC xenograft and patient-derived xenograft models, our previous study and others reported chemotherapy induction of the COX-2/PGE_2_ axis in promoting chemoresistance, via inducing compensatory proliferation of residual cancer stem cells^25^, and enhancing stemness via promoter methylation of let-7 and upregulation of the self-renewal gene SOX2^52^. Furthermore, in canine models of bladder cancer, COX-2 inhibition significantly enhanced chemotherapeutic response by ~40%^53^. Collectively, these prior studies and our current study convincingly show that pharmaceutical blockade of the COX-2/PGE_2_ axis could become a promising therapeutic approach in bladder cancer (in combination with chemotherapy and ICIs), since blockade of this axis has demonstrated consistent effects in targeting epithelial cancer stem cell repopulation, and reshaping the tumor microenvironment to allow CD8+ T-cell penetration into the tumor core. It was reported elsewhere that exogenous PGE_2_ contributes to a loss of pericyte coverage along the vasculature by breaking down pericyte-endothelial cell interactions^54^. Pharmaceutical blockade of the COX-2/PGE_2_ axis also restores pericyte-endothelial cell interactions to promote vasculature maturation (unpublished observations), which represents a plausible mechanism enabling more prominent T-cell penetration.

Furthermore, our results here draw an important mechanistic connection between tumor cell-derived COX-2/PGE_2_, immunogenic DC activation, and T-cell-inflamed tumors^55,56^. The importance of matured DCs in priming intratumoral T-cell activation has been demonstrated mechanistically in other cancer types^57,58^. For instance, DCs secrete CXCL9 and CXCL10 as chemoattractors for recruiting CXCR3+ T cells into the tumor microenvironment^59^. Intriguingly, blockade of PGE_2_ synthesis has been shown to increase the expression of CXCL9 and CXCL10 in murine models of cutaneous melanoma^51,60^, as well as our murine bladder cancer models (unpublished observations). However, the underlying mechanisms are not well studied. On the other hand, a lack of DC infiltration and maturation has been linked to defective CD8+ T-cell priming, leading to decreased IFNγ expression^61^. These studies have underscored the importance of DC presence in the tumor microenvironment as facilitators of antitumoral T-cell immunity. Nevertheless, the mechanisms of DC recruitment to the tumor bed and their activation are not fully understood. These recent studies underscore the importance of our current study and highlight the significance of tumor-derived PGE_2_ in regulating DC activation in MIBCs—in the chemotherapy treatment context—a notion that has not been clinically evaluated in this cancer type. Independently, these findings also highlight the concept to exploit drug-induced immunogenic cell death in bladder cancer. Although accumulating evidence has revealed a handful of chemotherapies exert beneficiary immunomodulatory effects by inducing immunogenic cell death in breast, colon, and non-small cell lung cancers^62–64^, the immunogenic effects of gemcitabine-cisplatin combination remained controversial and has not been fully elucidated until the current study. Indeed, unpublished observations by others indicate another more toxic chemotherapy regimen—i.e., MVAC (methotrexate, vinblastine, adriamycin, and cisplatin) seems to exert more clinical benefits than GC chemotherapy when combined with ICI in bladder cancer patients. Our unpublished observations revealed adriamycin (or doxorubicin) is likely responsible for the immunogenic effects of the MVAC chemotherapy combination, since adriamycin induces a relatively high membrane exposure of immunostimulatory DAMPs with a low iDAMP/PGE_2_ release into the tumor microenvironment.

Our findings pose “iDAMP blockade” as the next-generation drug combination to be evaluated with chemoimmunotherapy clinically, by converting immune-excluded tumors into T-cell-inflamed tumors when coadministered. Indeed, a new class of antagonists that target PGE_2_ receptors (EP) on downstream effector cells (e.g., dendritic cells or fibroblasts) might provide a next-generation approach for “iDAMP blockade”. However, it remains unclear whether this therapeutic combination would be more efficacious if chemoimmunotherapy was given in sequence^65^. New evidence from recent Phase 2 and 3 clinical trials suggested that a “switch maintenance” approach might provide superior clinical benefit when compared to the concurrent treatment approach^66,67^. In these trials, advanced bladder cancer patients that responded to or had stable disease after treatment with platinum-based chemotherapy were switched to maintenance immunotherapy, who demonstrated a superior overall (OS) and progression-free survival (PFS) compared to patients assigned to best supportive care. Similar results were obtained in stage III lung cancer patients that did not progress after platinum-based chemotherapy and were switched to maintenance immunotherapy^68^. These recent findings implicate that treatment sequence is another important determinant to a successful therapeutic response, that should be carefully considered.

Collectively, the current findings provide evidence that therapeutic targeting of COX-2 can be explored to convert immune-excluded into T-cell-inflamed bladder tumors; thereby, potentially expanding the response of MIBC patients towards chemoimmunotherapy. And thus, poses a promising strategy for future clinical evaluation.

## Methods

### Cell culture

The murine (FVB/NJ) bladder cancer cell lines, G69 and G7, were generated from K5.Stat3C transgenic mice^69^ exposed to N-butyl-N-(4-hydroxybutyl)-nitrosamine (BBN) via water intoxication for ≥15 weeks. Tumor-harboring bladders were harvested and dissociated to single-cell suspensions as previously described^24,25^. Established tumor cell lines were maintained in DMEM/F12 (Sigma, D6421) supplemented with 10% fetal bovine serum-containing D-glucose (Fisher, AAA168280E), 1× sodium pyruvate (Gibco, 160070), 1× glutamax (Gibco, 35050061), 1× insulin-transferrin-selenium (Gibco, 41400045), 1× Gentamicin (Gibco, 15710064). Bone-marrow-derived dendritic cells were maintained in RPMI media (Sigma, R8758) supplemented with 50 μM beta-mercaptoethanol (Santa Cruz Biotechnology, sc-202966), 1× glutamax (Gibco, 35050061), and 10% heat-inactivated fetal bovine serum. Cells were incubated at 37 °C in a humidified atmosphere containing 5% CO_2_. In vitro treatment of cancer cells with chemotherapy was carried out in DMEM high cultured media supplemented with reduced fetal bovine serum concentration at 2%. G69 and G7 cells were treated with IC50 concentrations of gemcitabine (TCI, 501332958) and cisplatin (Sigma, P4394). Celecoxib (Selleck Chemicals, 50-784-7) treatment of 3 μg/mL was used for all in vitro experiments. At the time of collection, cultured media were first centrifuged at 1200 × *g* for 5 min to ensure the collection of floating cells. Supernatants were then centrifuged at 18,000 × *g* for 15 min at 4 °C to pellet cellular debris. Debris-free supernatants were utilized for downstream ELISA and western blot analyses. Adherent cells were dissociated using TrypLE express enzyme (Gibco, 12605028), combined with the detached, floating cell pellets, and re-pelleted by centrifugation (1600 × *g* for 5 min at room temperature) for downstream flow cytometric and western blot analyses.

### Mice

All animal procedures were performed in ethical compliance and with approval by the Institutional Animal Care and Use Committees at Cedars-Sinai Medical Center. Wild-type male FVB/NJ mice at 6- to 8-week-old of age were utilized for all in vivo experiments. Mice have been housed in Cedars-Sinai Medical Center vivarium at ambient room temperature (22 ± 2 °C) with humidity of 40–70% and a light/dark cycle of 12 h/12 h.

### Tumor growth

G69 and G7 cells were harvested at their exponential growth phase (i.e., 70–80% confluency) using TrypLE express enzyme (Thermo, 12604013) and pelleted by centrifugation at 1200 × *g* for 5 min and washed once with DPBS (Sigma, D8537). 5 × 10^5^ cells were suspended in 20 uL of DPBS and injected into the left flank of mice. Tumor growth was recorded twice a week with caliper. Tumor volume was calculated using the standard formula (width × length × length/2). For tumor growth studies, the endpoint was 2 days after the last treatment unless mice were in moribund status, defined as signs of prolonged distress, tumor interfering with ambulation (normal movement), or access to food and water, or when tumor displayed signs of ulceration, or tumor size of >15 mm in diameter. The maximal tumor size was not exceeded.

### Treatment of G69 tumors with chemotherapy and anti-PD1 in vivo

Mice with established G69 or G7 tumors (i.e., tumor volume of ~50 mm^3^) were randomized into treatment groups (using RANDOM.com) and injected intraperitoneally (i.p.) with either vehicle (10% DMSO in 0.05% NaCl solution) or GC (gemcitabine at 120 mg/Kg; Fisher Scientific and cisplatin at 6 mg/Kg; Sigma–Aldrich) on day one and gemcitabine on day 8 to complete one cycle of chemotherapy. Mice were left to recover from chemotherapy for seven days upon which they received the second cycle of GC chemotherapy. Two days after the last gemcitabine treatment, tumors were harvested and processed for subsequent analyses. For the iDAMP blockade group, mice received celecoxib (10 mg/Kg; Sigma–Aldrich) daily until the completion of the experiment. For single therapy treatment, anti-PD1 antibody (200 ug/mouse, RMP1-14, Bio x Cell) was injected i.p. every other day for a total of three doses. For combination treatments, anti-PD1 was injected i.p. on days 9, 11, 13 of each chemotherapy cycle.

### Footpad vaccination assay

G69 cells were seeded at 4.7 × 10^4^ cells per mm^2^ and were treated with IC50 concentrations of cisplatin and gemcitabine with or without celecoxib for 24 h in vitro. After 24 h, adherent cells were washed with DPBS (Sigma, D8537) thoroughly, dissociated with TrypLE express enzyme, and pelleted by centrifugation (1300 × *g* for 5 min at room temperature). Cell pellets were washed with DPBS once more to ensure clearance of residual enzyme and pharmacological agent(s). 5 × 10^5^ cells were suspended in 15 μL of DPBS and were injected into the footpad of mice. Five days following vaccination mice were euthanized and the vaccine-draining lymph nodes (i.e., popliteal) were collected for subsequent flow cytometric analysis.

### CD8^+^ cell depletion

Mice were injected subcutaneously with aCD8 mAb (BioXcell, BE0004-1-A005) at a concentration of 200 μg per 30 mg mouse. Mice were injected every other day with the aCD8 mAb for the duration of the chemotherapy treatment. Efficacy of CD8-depletion was checked using murine tail-vain blood sampling coupled with flow cytometry analysis.

### Processing vaccine-draining lymph nodes for flow cytometry

Vaccine-draining lymph nodes were harvested 5 days post-vaccination of the murine footpad. Nondraining lymph nodes (i.e., opposite foot) were harvested as controls. Harvested lymph nodes were passed through a 70 μ mesh filter. Flow-through immune cells were then centrifuged. Lymph node cells were then collected, washed with DPBS, and processed for flow cytometric analysis.

### Generation of bone-marrow-derived dendritic cells

Generation of bone-marrow-derived dendritic cells followed a stepwise protocol previously described by Helft et al.^36^. In short, bone marrow cells were collected from the murine femur and tibia. In all, 10 × 10^6^ bone marrow cells were cultured in RPMI media (Sigma, R8758) supplemented with 50 μM beta-mercaptoethanol (Santa Cruz Biotechnology, sc-202966), 10% heat-inactivated fetal bovine serum (Thermo, A3840001), and 20 ng/mL of GM-CSF (Miltenyi,130-094-043) for 2 days. On day 2, half of the media was discarded and new fully supplemented media with GM-CSF (2×, 40 ng/ml) was added to cultures. On day 3 the entire culture media was discarded and replaced with fresh fully supplemented media with GM-CSF (20 ng/mL) until day 6.

### Treating BMDCs with cultured media

Non-adherent BMDCs in the culture supernatant and loosely adherent BMDCs from 6 days of culture were harvested using 5 μM EDTA solution. 1 × 10^5^ cells were suspended in GC pre-treated G69 cultured media (20% of total volume and 80% base DC media as described above) in the absence or presence of iDAMP blockade. Cultured media PGE_2_ sequestration was achieved by treating the cultured media with 100 nM of anti-PGE_2_ mAb (Cayman, 10009814) for at least 30 min on ice. LPS (InvivoGen; tlrl-3pelps; 100 ng/ml) treatment was used as a positive control. BMDCs were cultured and collected at 24-h post-activation with appropriate culture medium treatment.

### Immunofluorescence analysis

Formalin-fixed paraffin-embedded G69 and G7 tumor tissues were deparaffinized in xylene baths twice for 5 min and rehydrated using an ethanol gradient (2 × 100%, 95%, and 70%) for 50 dips. Rehydrated tissues were incubated in neutral formalin for 20 min to ensure tissue adhesion. Antigen retrieval was performed in Tris/EDTA buffer with 10% glycerol at pH 9 for 20 min in a vegetable steamer. Tissue sections were blocked with animal-free blocking solution (Cell Signaling Technologies, 15019) for 10 min and incubated with primary antibodies against CD3 (Abcam, ab5690, 1:200), COX-2 (Cell Signaling Technologies, 12282 S, 1:200), CD11c(Cell Signaling Technologies, 97585, 1:200) and aSMA (Abcam, ab184705, 1:1000) for 2 h at room temperature (RT). Tissues were washed in TBS with 0.05% Tween-20 (TBST) and incubated with HRP polymer-conjugated secondary antibodies for 15 min at RT (Goat Anti-Mouse IgG H&L HRP polymer, Abcam, ab214879, prediluted; Goat Anti-Rabbit IgG H&L HRP polymer, Abcam, ab214880, prediluted). Tyramide signal amplification and nuclei counterstining were performed by incubating tissues with OPAL 520 (Akoya Biosciences, FP1487001KT, 1:500) and OPAL 650 (Akoya Biosciences, FP1496001KT, 1:500) fluorophores for 10 min at RT, washing with TBST and counterstaining with DAPI (2 ug/mL) for 5 min. Tissues were mounted using ProLong Gold Antifade (Thermo, P36930). Images were acquired using Zeiss Apotome upright microscope equipped with Axiocam monochrome camera.

### Quantification of CD3+ T-cell density

Immunofluorescence images were acquired using Zeiss Axio Imager 2 at ×20 magnification and analyzed using Zen software (version 3.3, blue edition). Regions of interest (ROI’s) were defined randomly from the whole tissue section and 3 to 5 ROI’s were selected per tumor tissue section. CD3+ cells were defined based on CD3 and DAPI positive co-staining. Immune cell infiltration was determined by performing linear regression, comparing the density of CD3+ cells within each ROI to the normalized mean distance of the ROI to the tumor periphery. The density of CD3+ cells was determined by calculating the number of CD3+ cells per area of ROI. The mean distance of the ROI to the tumor periphery was determined by calculating the mean nearest distance of the ROI border to the tumor periphery. Mean distances between samples were then normalized based on the maximum distance between the tumor core and tumor periphery^19^.

### Flow cytometry/FACS

All immune cell samples were suspended in 50 μL of anti-CD16/32 antibody (Biolegend, 101320, 1:200) solution in PBS for 10 min on ice prior to subsequent immunostaining. Tumor-infiltrating lymphocytes were stained with the following antibodies: CD45-BV570 (Biolegend, 103135, 1:400); CD3-PE (Biolegend, 100206, 1:200); CD4-APC (Biolegend, 100411, 1:200); and CD8-FITC (Biolegend, 100706, 1:200), Tbet-BV711 (Biolegend, 644819, 1:200), IFNg-BV785 (Biolegend, 505838), and GZMb-AF647 (Biolegend, 515405, 1:200). Intracellular staining was performed using the BD Cytofix/Cytoperm (BD, 554714) following the manufacturer’s protocol.Vaccine-vdLN dendritic cells were stained with the following antibodies: CD45-BV570 (Biolegend, 103135, 1:400), CD11c-PE/Cy7 (Biolegend, 117318, 1:200); MHCII-BV510 (Fisher, 50402975, 1:400); H2Kq-AF647 (Biolegend, 115106, 1:200), CD40-FITC (Biolegend, 124608, 1:200), CD86-PerCP/Cy5.5 (Biolegend, 105028, 1:200). Bone-marrow-derived dendritic cells were stained with: CD45-BV570 (Biolegend, 103135, 1:400); CD11c-PE/Cy7 (Biolegend, 117318, 1:200); MHCII-BV510 (Fisher, 50402975, 1:400); H2Kq-AF647 (Biolegend, 115106, 1:200), CD40-FITC (Biolegend, 124608, 1:200), CD86-PerCP/Cy5.5 (Biolegend, 105028, 1:200), IL10-BV421 (Biolegend, 505022, 1:200), IL12-PE (Biolegend, 505204, 1:200), and Live/Dead-NearIR stain (Thermo, L10119, 1:2000). To determine cell-surface DAMP expression, chemotherapy-treated cancer cells were stained with anti-CRT-PE (Cell Signaling Technologies, 19780 S, 1:200) or anti-HSP70-PE (Miltenyi, 130-105-549, 1:200). Concurrently, these DAMP-stained cells were labeled with a cell viability dye (DAPI at a 3 μM concentration) for dead cell exclusion. All antibody cocktails were diluted in ice-cold FACS buffer or Briliant Stain buffer (BD, 563794) when more than one brillian violet-conjugated antibody was used. Cells were incubated with the appropriate antibody cocktails on ice for 20 min and washed thoroughly with ice-cold FACS buffer. Flow cytometric analysis of immune cells was performed on BD LSRFortessa™ and Cytek™ Northern Lights cell analyzers. Flow cytometric analysis of cell-surface CRT and HSP70 was performed on BD LSRFortessa™. All flow cytometry data were processed using FlowJo software v.10.7.1.

### ELISA

ELISA analysis of PGE2 (Cayman, 514010) and HMGB1 (Arigo, ARG81310) released by cancer cells and serum from G69 and G7 tumor-bearing mice was performed according to the manufacturer’s protocol.

### Western blot

Cell pellets were lysed in RIPA buffer (Emd Millipore, 20–188) with complete protease (Sigma, 11836153001) and phosphatase (Sigma, 04906837001) inhibitor cocktails. Lysates were collected by centrifugation, 18,000 × *g* for 15 min at 4 °C. Protein concentration was measured using Bradford BCA colorimetric assay (Bio-Rad, 5000006). In all, 15 µg of sample lysates were subjected to western blot analysis using 10% Tris-Glycine gel under reducing conditions. Proteins were transferred onto PVDF membranes (Emd Millipore, IPVH00010) and probed with the following primary antibodies: COX-2 [74 kDa] at 1:1000 (Cell Signaling Technologies, 12282 S); GAPDH [~37 kDa] at 1:2000 (Santa Cruz biotechnology, SC-32233); and HMGB1 [~29 kDa] at 1:1000 (Biolegend, 651402). Secondary antibodies were purchased from the following sources: anti-mouse-HRP at 1:10,000 (Boster, BA1075) and anti-rabbit-HRP at 1:10,000 (Cell Signaling Technologies, 7074 S). Western blot bands were visualized using an enhanced chemiluminescence system (Thermo, 32106) on the iBright™ CL750 system and iBright Analysis Software version 1.5.0.

### PTGS2 expression in ICB-treated bladder cancer patients

PTGS2 expression data were obtained from RNA-seq analysis of patient samples of a large phase II clinical trial (IMvigor210) that investigated the clinical activity of Atezolizumab (PD-L1 monoclonal antibody) in patients with locally advanced and metastatic urothelial carcinoma^41^. There were 68 samples from patients classified as complete or partial responders (CR/PR) and 230 samples from patients classified as having a stable disease or progressive disease (SD/PD). Two sample T-test was performed to compare PTGS2 gene expression between the CR/PR and SD/PD groups using the normalized RNA counts.

### Statistics and reproducibility

All data were evaluated and graphed using Prism GraphPad ver. 9 software. Statistical comparison between control and experimental groups was performed utilizing two-tailed Student’s *t-test*, one-way analysis of variance (ANOVA) with multiple comparisons test when appropriate. Quantified data shown were repeated at least three times in independent experiments (unless specified differently). Data shown are represented as mean ± SEM. *P*-value < 0.05 was considered statistically significant.

### Reporting summary

Further information on research design is available in the Nature Research Reporting Summary linked to this article.

## Supplementary information

Supplementary Information

Reporting Summary

## Data Availability

The Mariathasan et al.^19^ data used in this study are available in the European Genome Phenome Archive under accession number EGAS00001002556. In addition, source code and processed data used for all analyses presented in Mariathasan et al.^19^ are available in IMvigor210CoreBiologies and can be downloaded from [http://research-pub.gene.com/IMvigor210CoreBiologies]. All the source data and uncropped scans of blots supporting the findings of this study are provided with this paper as Source data file. The remaining data are available within the Article and Supplementary Information. Source data are provided with this paper.

## Source data

Source Data

## References

[1] Bray F (2018). Global cancer statistics 2018: GLOBOCAN estimates of incidence and mortality worldwide for 36 cancers in 185 countries. CA Cancer J. Clin..

[2] Grossman HB (2003). Neoadjuvant chemotherapy plus cystectomy compared with cystectomy alone for locally advanced bladder cancer. N. Engl. J. Med..

[3] Pectasides, D., Pectasides, M. & Nikolaou, M. Adjuvant and neoadjuvant chemotherapy in muscle invasive bladder cancer: Literature review. *Eur. Urol.*10.1016/j.eururo.2005.03.025 (2005). 10.1016/j.eururo.2005.03.025 15967253

[4] Yin M (2016). Neoadjuvant chemotherapy for muscle‐invasive bladder cancer: a systematic review and two‐step meta‐analysis. Oncologist.

[5] Meeks, J. J. et al. A systematic review of neoadjuvant and adjuvant chemotherapy for muscle-invasive bladder cancer. *Eur. Urol.*10.1016/j.eururo.2012.05.048 (2012). 10.1016/j.eururo.2012.05.048 22677572

[6] Green DR, Ferguson T, Zitvogel L, Kroemer G (2009). Immunogenic and tolerogenic cell death. Nat. Rev. Immunol..

[7] Krysko, D. V. et al. Immunogenic cell death and DAMPs in cancer therapy. *Nat. Rev. Cancer*10.1038/nrc3380 (2012). 10.1038/nrc3380 23151605

[8] Kroemer, G., Galluzzi, L., Kepp, O. & Zitvogel, L. Immunogenic cell death in cancer therapy. *Annu. Rev. Immunol*. 10.1146/annurev-immunol-032712-100008 (2013). 10.1146/annurev-immunol-032712-100008 23157435

[9] Vanmeerbeek, I. et al. Trial watch: chemotherapy-induced immunogenic cell death in immuno-oncology. *OncoImmunology*10.1080/2162402X.2019.1703449 (2020). 10.1080/2162402X.2019.1703449 PMC6959434 32002302

[10] Galluzzi, L. et al. Consensus guidelines for the definition, detection and interpretation of immunogenic cell death. *J. Immunother. Cancer*10.1136/jitc-2019-000337 (2020). 10.1136/jitc-2019-000337 PMC7064135 32209603

[11] Hayashi K (2020). Tipping the immunostimulatory and inhibitory DAMP balance to harness immunogenic cell death. Nat. Commun.

[12] Hayashi, K., Nikolos, F. & Chan, K. S. Inhibitory DAMPs in immunogenic cell death and its clinical implications. *Cell Stress*10.15698/cst2021.04.247 (2021). 10.15698/cst2021.04.247 PMC8012883 33821233

[13] Bracci L, Schiavoni G, Sistigu A, Belardelli F (2014). Immune-based mechanisms of cytotoxic chemotherapy: Implications for the design of novel and rationale-based combined treatments against cancer. Cell Death Differ..

[14] Fridman WH, Zitvogel L, Sautès-Fridman C, Kroemer G (2017). The immune contexture in cancer prognosis and treatment. Nat. Rev. Clin. Oncol..

[15] Sweis, R. F. et al. Molecular drivers of the non- T-cell-inflamed tumor microenvironment in urothelial bladder cancer. *Cancer Immunol. Res*. 10.1158/2326-6066.CIR-15-0274 (2016). 10.1158/2326-6066.CIR-15-0274 PMC4943758 27197067

[16] Saito R (2018). Molecular subtype-specific immunocompetent models of high-grade urothelial carcinoma reveal differential neoantigen expression and response to immunotherapy. Cancer Res.

[17] Galsky MD (2020). Atezolizumab with or without chemotherapy in metastatic urothelial cancer (IMvigor130): a multicentre, randomised, placebo-controlled phase 3 trial. Lancet.

[18] Powles, T. et al. Pembrolizumab alone or combined with chemotherapy versus chemotherapy as first-line therapy for advanced urothelial carcinoma (KEYNOTE-361): a randomised, open-label, phase 3 trial. *Lancet Oncol*. 10.1016/S1470-2045(21)00152-2 (2021). 10.1016/S1470-2045(21)00152-2 34051178

[19] Mariathasan, S. et al. TGFβ attenuates tumour response to PD-L1 blockade by contributing to exclusion of T cells. *Nature*10.1038/nature25501 (2018). 10.1038/nature25501 PMC6028240 29443960

[20] Luke, J. J., Bao, R., Sweis, R. F., Spranger, S. & Gajewski, T. F. WNT/b-catenin pathway activation correlates with immune exclusion across human cancers. *Clin. Cancer Res*. 10.1158/1078-0432.CCR-18-1942 (2019). 10.1158/1078-0432.CCR-18-1942 PMC6522301 30635339

[21] John BA, Said N (2017). Insights from animal models of bladder cancer: recent advances, challenges, and opportunities. Oncotarget.

[22] Fantini D (2018). A Carcinogen-induced mouse model recapitulates the molecular alterations of human muscle invasive bladder cancer. Oncogene.

[23] Kelsey R (2018). Bladder cancer: BBN mouse model mimics human MIBC*.*. Nat. Rev. Urol..

[24] Ho PL, Lay EJ, Jian W, Parra D, Chan KS (2012). Stat3 activation in urothelial stem cells leads to direct progression to invasive bladder cancer. Cancer Res..

[25] Kurtova AV (2015). Blocking PGE2-induced tumour repopulation abrogates bladder cancer chemoresistance. Nature.

[26] Lee YC (2019). Collagen-rich airway smooth muscle cells are a metastatic niche for tumor colonization in the lung. Nat. Commun.

[27] Chen DS, Mellman I (2017). Elements of cancer immunity and the cancer-immune set point. Nature.

[28] Eckstein, M. et al. Cytotoxic T-cell-related gene expression signature predicts improved survival in muscle-invasive urothelial bladder cancer patients after radical cystectomy and adjuvant chemotherapy. *J. Immunother. Cancer*10.1136/jitc-2019-000162 (2020). 10.1136/jitc-2019-000162 PMC7253053 32448798

[29] St. Paul M, Ohashi PS (2020). The roles of CD8+ T cell subsets in antitumor immunity. Trends Cell Biol..

[30] Fu C, Jiang A (2018). Dendritic cells and CD8 T cell immunity in tumor microenvironment. Front. Immunol..

[31] Wculek SK (2020). Dendritic cells in cancer immunology and immunotherapy. Nat. Rev. Immunol..

[32] Steinman RM (1991). The dendritic cell system and its role in immunogenicity. Annu. Rev. Immunol..

[33] Itakura E (2011). IL-10 expression by primary tumor cells correlates with melanoma progression from radial to vertical growth phase and development of metastatic competence. Mod. Pathol..

[34] Kratky W, Reis E Sousa C, Oxenius A, Spörri R (2011). Direct activation of antigen-presenting cells is required for CD8+ T-cell priming and tumor vaccination. Proc. Natl Acad. Sci. USA.

[35] Galluzzi L, Buqué A, Kepp O, Zitvogel L, Kroemer G (2015). Immunological effects of conventional chemotherapy and targeted anticancer agents. Cancer Cell.

[36] Helft J (2015). GM-CSF mouse bone marrow cultures comprise a heterogeneous population of CD11c+MHCII+ macrophages and dendritic cells. Immunity.

[37] Humeau, J., Lévesque, S., Kroemer, G. & Pol, J. G. In *Methods in Molecular Biology*, vol. 1884, 297–315 (Humana Press Inc., 2019). 10.1007/978-1-4939-8885-3_21 30465212

[38] Bellmunt J, Powles T, Vogelzang NJ (2017). A review on the evolution of PD-1/PD-L1 immunotherapy for bladder cancer: the future is now. Cancer Treat. Rev..

[39] Charles A Janeway, J., Travers, P., Walport, M. & Shlomchik, M. J. The course of the adaptive response to infection. https://www.ncbi.nlm.nih.gov/books/NBK27125 (2001).

[40] Chan KS (2016). Molecular pathways: targeting cancer stem cells awakened by chemotherapy to abrogate tumor repopulation. Clin. Cancer Res..

[41] Rosenberg JE (2016). Atezolizumab in patients with locally advanced and metastatic urothelial carcinoma who have progressed following treatment with platinum-based chemotherapy: A single-arm, multicentre, phase 2 trial. Lancet.

[42] Tumeh PC (2014). PD-1 blockade induces responses by inhibiting adaptive immune resistance. Nature.

[43] Schmid P (2020). Atezolizumab plus nab-paclitaxel as first-line treatment for unresectable, locally advanced or metastatic triple-negative breast cancer (IMpassion130): updated efficacy results from a randomised, double-blind, placebo-controlled, phase 3 trial. Lancet Oncol..

[44] West H (2019). Atezolizumab in combination with carboplatin plus nab-paclitaxel chemotherapy compared with chemotherapy alone as first-line treatment for metastatic non-squamous non-small-cell lung cancer (IMpower130): a multicentre, randomised, open-label, phase 3 trial. Lancet Oncol..

[45] Gandhi L (2018). Pembrolizumab plus chemotherapy in metastatic non–small-cell lung cancer. N. Engl. J. Med..

[46] Burtness B (2019). Pembrolizumab alone or with chemotherapy versus cetuximab with chemotherapy for recurrent or metastatic squamous cell carcinoma of the head and neck (KEYNOTE-048): a randomised, open-label, phase 3 study. Lancet.

[47] Galsky MD (2018). Phase 2 trial of gemcitabine, cisplatin, plus ipilimumab in patients with metastatic urothelial cancer and impact of dna damage response gene mutations on outcomes. Eur. Urol..

[48] Lee, Y. et al. The dynamic roles of the bladder tumor microenvironment (bTME). *Nat. Rev. Urol*. *In Press*10.1038/s41585-022-00608-y PMC10112172 35764795

[49] Oresta, B. et al. Mitochondrial metabolic reprogramming controls the induction of immunogenic cell death and efficacy of chemotherapy in bladder cancer. *Sci. Transl. Med*. 10.1126/SCITRANSLMED.ABA6110 (2021). 10.1126/scitranslmed.aba6110 33408185

[50] Garris, C. S. & Luke, J. J. Dendritic cells, the T-cell-inflamed tumor microenvironment, and immunotherapy treatment response. *Clin Cancer Res.* **26**, 3901–3907. 10.1158/1078-0432.CCR-19-1321 (2020). 10.1158/1078-0432.CCR-19-1321 PMC7607412 32332013

[51] Zelenay, S. et al. Cyclooxygenase-dependent tumor growth through evasion of immunity. *Cell* **10**, 1257–1570 (2015). 10.1016/j.cell.2015.08.015 PMC4597191 26343581

[52] Ooki A (2018). YAP1 and COX2 coordinately regulate urothelial cancer stem-like cells. Cancer Res..

[53] Knapp DW (2016). A nonselective cyclooxygenase inhibitor enhances the activity of vinblastine in a naturally-occurring canine model of invasive urothelial carcinoma. Bladder Cancer.

[54] Perrot CY, Herrera JL, Fournier-Goss AE, Komatsu M (2020). Prostaglandin E2 breaks down pericyte–endothelial cell interaction via EP1 and EP4-dependent downregulation of pericyte N-cadherin, connexin-43, and R-Ras. Sci. Rep..

[55] Barry KC (2018). A natural killer–dendritic cell axis defines checkpoint therapy–responsive tumor microenvironments. Nat. Med..

[56] Mayoux M (2020). Dendritic cells dictate responses to PD-L1 blockade cancer immunotherapy. Sci. Transl. Med..

[57] Salmon H (2016). Expansion and activation of CD103+ dendritic cell progenitors at the tumor site enhances tumor responses to therapeutic PD-L1 and BRAF inhibition. Immunity.

[58] Hildner K (2008). Batf3 deficiency reveals a critical role for CD8α+ dendritic cells in cytotoxic T cell immunity. Science.

[59] Spranger S, Dai D, Horton B, Gajewski TF (2017). Tumor-residing Batf3 dendritic cells are required for effector T cell trafficking and adoptive T cell therapy. Cancer Cell.

[60] Kim SH (2019). The COX2 effector microsomal PGE2 synthase 1 is a regulator of immunosuppression in cutaneous melanoma. Clin. Cancer Res..

[61] Sánchez-Paulete AR (2016). Cancer immunotherapy with immunomodulatory anti-CD137 and anti–PD-1 monoclonal antibodies requires BATF3-dependent dendritic cells. Cancer Discov..

[62] Tesniere A (2010). Immunogenic death of colon cancer cells treated with oxaliplatin. Oncogene.

[63] Hato SV, Khong A, De Vries IJM, Lesterhuis WJ (2014). Molecular pathways: the immunogenic effects of platinum-based chemotherapeutics. Clin. Cancer Res..

[64] Apetoh L (2007). Toll-like receptor 4-dependent contribution of the immune system to anticancer chemotherapy and radiotherapy. Nat. Med..

[65] Zengin ZB, Meza L, Pal SK, Grivas P (2021). Chemoimmunotherapy in urothelial cancer: concurrent or sequential?. Lancet Oncol..

[66] Powles T (2020). Avelumab maintenance therapy for advanced or metastatic urothelial carcinoma. N. Engl. J. Med..

[67] Galsky MD (2020). Randomized double-blind phase II study of maintenance pembrolizumab versus placebo after first-line chemotherapy in patients with metastatic urothelial cancer. J. Clin. Oncol..

[68] Antonia SJ (2017). Durvalumab after chemoradiotherapy in stage III non–small-cell lung cancer. N. Engl. J. Med..

[69] Sano S (2005). Stat3 links activated keratinocytes and immunocytes required for development of psoriasis in a novel transgenic mouse model. Nat. Med..

