# Supplementary material for: Cell death-induced immunogenicity enhances chemoimmunotherapeutic response by converting immune-excluded into T-cell inflamed bladder tumors
Source: Nat Commun. 2022 Mar 28;13:1487. doi: 10.1038/s41467-022-29026-9 (PMC8960844; doi:10.1038/s41467-022-29026-9)
Supplement: Supplementary file 1 — Supplementary Information [file 41467_2022_29026_MOESM1_ESM.pdf]

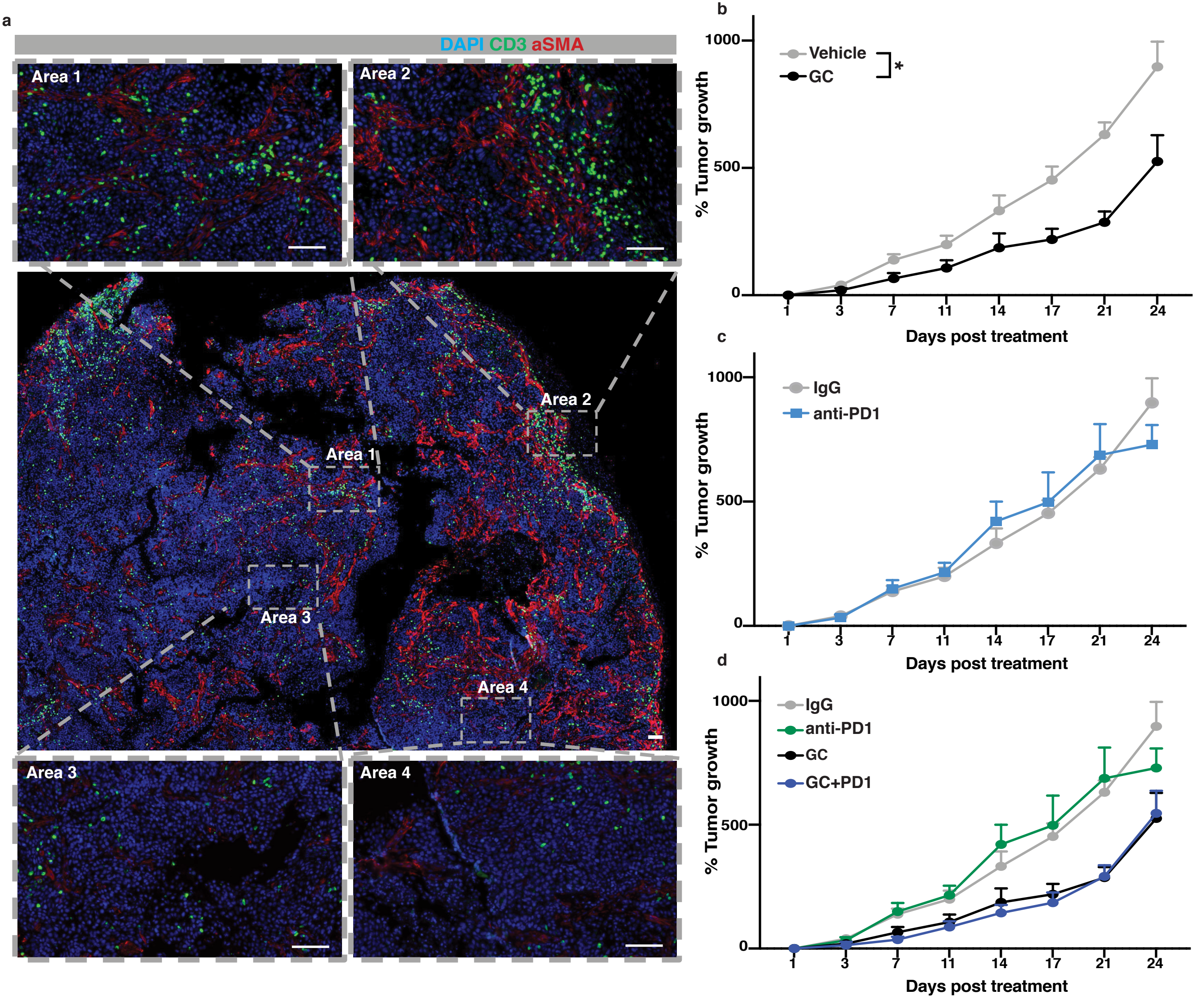

Supplementary Figure 1. G7 tumors are resistance to both chemotherapy/immunotherapy and their combonation. (a) Immunofluorescence analysis of CD3+ T cell infiltration and aSMA expression in G7 treatment-naïve tumor tissues (Scale bar: 100 µm; Images representative of n=3 independent experiments). (b-d) Percent growth curves of G7 tumors from vehicle (n=5) and GC (n=8), anti-PD1 (n=5) and GC+anti-PD1 (n=8) upon two cycles of chemotherapy. Percent tumor growth was obtained by normalizing to starting tumor volumes. Data are presented as mean values +/- SEM. (b: Two-tailed, unpaired t test at endpoint. GC vs Vehicle p=0.036). Source data are provided as a Source data file.

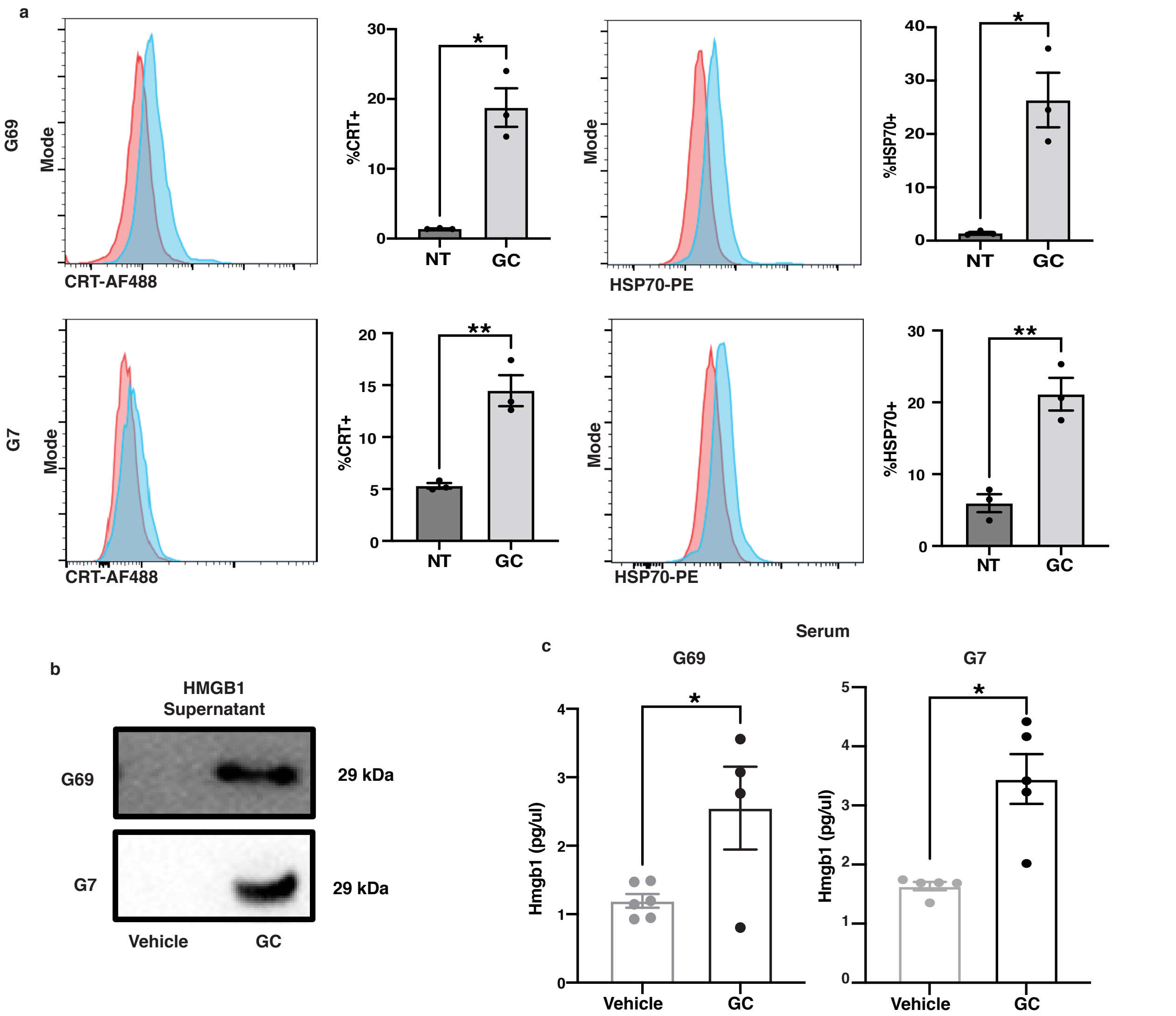

Supplementary Figure 2. GC induces the release of bona-fide immunostimulatory DAMPs both in vivo and in vitro. (a) Histograms and quantification of CRT and HSP70 expression by flow cytometry in G69 (top; n=3 biologically independent samples; Statisitcs: Two-tailed, unapired t test with Welch's correction. CRT:p=0.024; HSP70:p=0.039) and G7 (bottom; n=3 biologically independent samples. Statisitcs: Two-tailed, unapired t test. CRT:p=0.004; HSP70:p=0.004) cells treated with either vehicle or GC for 48 hours. (b) Western blot analysis of HMGB1 release in the culture media of G69 (top) or G7 (bottom) cells treated with either vehicle or GC for 48 hours (n=3 biologically independent experiments). (c) ELISA for HMGB1 release in the serum of G69 (top, n=6 & n=4 biologically independent samples for vehicle and GC respectively. Two-tailed, unpaired t test p=0.04) or G7-tumor bearing mice (bottom; n=5 biologically independent samples. Two-tailed, unpaired t test with Welch's correction p=0.017) treated with either vehicle or GC for 2 cycles. Source data are provided as a Source data file.

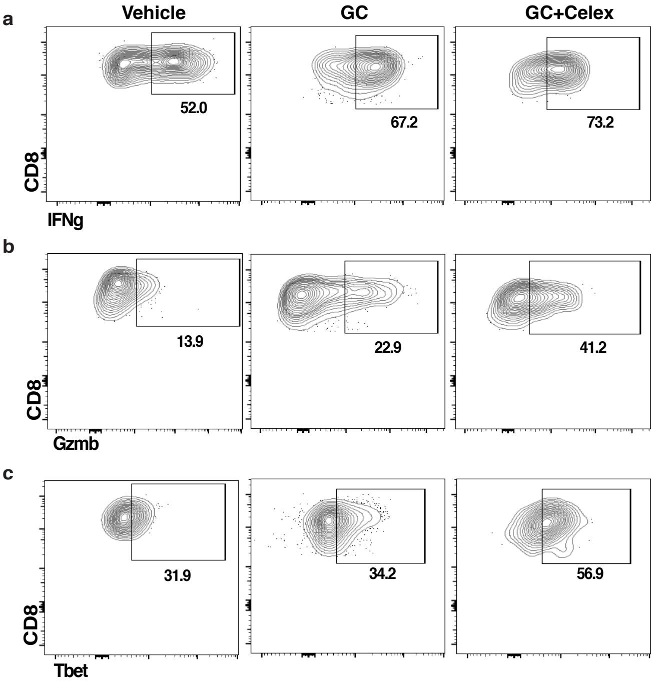

Supplementary Figure 3. iDAMP blockade induces Tc1 differentiation in CD8+ TILs (**a-c**) Contour plots of IFNγ, GZMb and Tbet expression in CD8+ TILs from G69 tumor tissues treated with either Vehicle, GC, or GC+Celex for 2 cycles (n=3 biologically independent experiments).
