# Supplementary material for: Cell death-induced immunogenicity enhances chemoimmunotherapeutic response by converting immune-excluded into T-cell inflamed bladder tumors
Source: Nat Commun. 2022 Mar 28;13:1487. doi: 10.1038/s41467-022-29026-9 (PMC8960844; doi:10.1038/s41467-022-29026-9)
Supplement: Supplementary file 2 — Reporting Summary [file 41467_2022_29026_MOESM2_ESM.pdf]

nature research

Corresponding author(s): Keith Syson Chan

Last updated by author(s): Feb 16, 2022

Reporting Summary

Nature Research wishes to improve the reproducibility of the work that we publish. This form provides structure for consistency and transparency in reporting. For further information on Nature Research policies, see our Editorial Policies and the Editorial Policy Checklist.

Statistics

For all statistical analyses, confirm that the following items are present in the figure legend, table legend, main text, or Methods section.

n/a	Confirmed	
☐	☒	The exact sample size (*n*) for each experimental group/condition, given as a discrete number and unit of measurement
☐	☒	A statement on whether measurements were taken from distinct samples or whether the same sample was measured repeatedly
☐	☒	The statistical test(s) used AND whether they are one- or two-sided<br>*Only common tests should be described solely by name; describe more complex techniques in the Methods section.*
☒	☐	A description of all covariates tested
☐	☒	A description of any assumptions or corrections, such as tests of normality and adjustment for multiple comparisons
☐	☒	A full description of the statistical parameters including central tendency (e.g. means) or other basic estimates (e.g. regression coefficient) AND variation (e.g. standard deviation) or associated estimates of uncertainty (e.g. confidence intervals)
☐	☒	For null hypothesis testing, the test statistic (e.g. *F*, *t*, *r*) with confidence intervals, effect sizes, degrees of freedom and *P* value noted<br>*Give P values as exact values whenever suitable.*
☒	☐	For Bayesian analysis, information on the choice of priors and Markov chain Monte Carlo settings
☒	☐	For hierarchical and complex designs, identification of the appropriate level for tests and full reporting of outcomes
☒	☐	Estimates of effect sizes (e.g. Cohen's *d*, Pearson's *r*), indicating how they were calculated

Our web collection on statistics for biologists contains articles on many of the points above.

Software and code

Policy information about availability of computer code

Data collection	BD LSR Fortessa; Cytek Northern Lights; iBright CL750, Zeiss Axio Imager 2
Data analysis	FlowJo v10.7.1; Prism Graphpad Version 9; iBright Analysis Software version 1.5.0, Zen software (version 3.3, blue edition)

For manuscripts utilizing custom algorithms or software that are central to the research but not yet described in published literature, software must be made available to editors and reviewers. We strongly encourage code deposition in a community repository (e.g. GitHub). See the Nature Research guidelines for submitting code & software for further information.

Data

Policy information about availability of data

All manuscripts must include a data availability statement. This statement should provide the following information, where applicable:
- Accession codes, unique identifiers, or web links for publicly available datasets
- A list of figures that have associated raw data
- A description of any restrictions on data availability

The Mariathasan et al.data used in this study are available in the European Genome Phenome Archive under accession number EGAS00001002556 [https://ega-archive.org/studies/EGAS00001002556]. In addition, source code and processed data used for all analyses presented in Mariathasan et al. are available in IMvigor210CoreBiologies and can be downloaded from [http://research-pub.gene.com/IMvigor210CoreBiologies]. All the source data and uncropped scans of blots supporting the findings of this study are provided with this paper as Source data file. The remaining data are available within the Article and Supplementary Information.

Field-specific reporting

Please select the one below that is the best fit for your research. If you are not sure, read the appropriate sections before making your selection.

[x] Life sciences [ ] Behavioural & social sciences [ ] Ecological, evolutionary & environmental sciences

For a reference copy of the document with all sections, see nature.com/documents/nr-reporting-summary-flat.pdf

Life sciences study design

All studies must disclose on these points even when the disclosure is negative.

Sample size	Sample size was calculated using a pilot chemotherapy assay: with a two-tailed alpha of 0.05 and power of 90%, each treatment group required at least 5 mice
Data exclusions	No data were excluded from the analysis
Replication	The data presented (if not biologically replicated), were replicated three times (at least) independently. The data provided in the manuscript are representative and reflect the reproducibility of the experiments. All replication attempts were successful.
Randomization	Mice were initially randomized using an online software prior to treatment
Blinding	Measurements of tumors were conducted by a blinded researcher that had no prior knowledge of treatment group stratifications. Another researcher was blinded to the treatment groups when quantifying immunofluorescence stained immune cells in the animal experiments. For in vitro cell culture studies blinding was not relevant as all measures were quantified by standard cellular or biochemical assays.

Reporting for specific materials, systems and methods

We require information from authors about some types of materials, experimental systems and methods used in many studies. Here, indicate whether each material, system or method listed is relevant to your study. If you are not sure if a list item applies to your research, read the appropriate section before selecting a response.

Materials & experimental systems

n/a	Involved in the study	
[ ]	[x]	Antibodies
[ ]	[x]	Eukaryotic cell lines
[x]	[ ]	Palaeontology and archaeology
[ ]	[x]	Animals and other organisms
[x]	[ ]	Human research participants
[x]	[ ]	Clinical data
[x]	[ ]	Dual use research of concern

Methods

n/a	Involved in the study	
[x]	[ ]	ChIP-seq
[ ]	[x]	Flow cytometry
[x]	[ ]	MRI-based neuroimaging

Antibodies

Antibodies used

anti-CD45-BV570 (Biolegend, 103135, 1:400),
anti-CD11c-PE/Cy7 (Biolegend, 117318, 1:200),
anti-MHCII-BV510 (Biolegend, 107636, 1:400),
anti-H2Kq-AF647 (Biolegend, 115106, 1:200),
anti-CD40-FITC (Biolegend, 124608, 1:200),
anti-CD86-PerCP/Cy5.5 (Biolegend, 105028, 1:200),
anti-CD3e-PE (Biolegend, 100206, 1:200),
anti-CD4-APC (Biolegend, 100411, 1:200),
anti-CD8a-FITC (Biolegend, 100706, 1:200),
anti-IFNg-BV785 (Biolegend, 505838, 1:200),
anti-GZMb-AF647 (Biolegend, 515405, 1:200),
anti-Tbet-BV711 (Bioloegend, 644819, 1:200),
anti-IL-10-BV421 (Biolegend, 505022, 1:200),
anti-IL-12-PE (Biolegend, 505204, 1:200),
Streptavidin-PE/Cy5 (Biolegend, 405205, 1:500),
Live/Dead-NearIR stain (Thermo, L10119, 1:2000),
DAPI (Biolegend, 422801, 3uM)
anti-CRT-PE (Cell Signaling, 19780S, 1:200),
anti-CRT (Cell Signaling, 12238, 1:1000),

anti-HSP70-PE (Miltenyi, 130-105-549, 1:200),
anti-COX-2 (Cell Signaling, 12282S, 1:1,000 Western, 1:200 Immunofluorescence),
anti-GAPDH (Santa Cruz biotechnology, SC-32233, 1:2,000),
anti-CD3 (Abcam, ac16669, 1:200),
anti-CD11c (Cell Signaling Technologies, 97585 1:200),
anti-aSMA (Abcam, ab5694, 1:1000),
anti-HMGB1 (Biolegend, 651402, 1:1,000),
anti-mouse-HRP (Boster, BA1075, 1:10,000),
anti-rabbit-HRP (Cell Signaling, 7074S, 1:10,000),
anti-CD16/32 antibody (Biolegend, 101320, 1:200),
anti-Mouse IgG H&L HRP polymer (Abcam, ab214879, prediluted)
anti-Rabbit IgG H&L HRP polymer (Abcam, ab214879, prediluted)

Validation

All primary antibodies were confirmed on the species and application through the validation statement on the manufacturer's website and their use in the literature. We are providing here the list of antibodies used.
anti-CD45-BV570 (Biolegend, 103135) validated by the company (https://www.biolegend.com/en-us/products/brilliant-violet-570-anti-mouse-cd45-antibody-7452) and by users (cited 11 times)
anti-CD11c-PE/Cy7 (Biolegend, 117318) validated by the company (https://www.biolegend.com/en-us/products/pe-cyanine7-anti-mouse-cd11c-antibody-3086) and by users (cited 137 tmes)
anti-MHCII-BV510 (Biolegend, 107636) validated by the company (https://www.biolegend.com/en-us/products/brilliant-violet-510-anti-mouse-i-a-i-e-antibody-7997) and users (cited 16 times)
anti-H2Kq-AF647 (Biolegend, 115106) validated by the company (https://www.biolegend.com/en-us/products/alexa-fluor-647-anti-mouse-h-2kq-antibody-4136) and users (cited 4 times)
anti-CD40-FITC (Biolegend, 124608) validated by the company (https://www.biolegend.com/en-us/products/fitc-anti-mouse-cd40-antibody-4982) and users (cited 13 times)
anti-CD86-PerCP/Cy5.5 (Biolegend, 105028) validated by the company (https://www.biolegend.com/en-us/products/percp-cyanine5-5-anti-mouse-cd86-antibody-4276) and users (cited 11 times)
anti-CD3e-PE (Biolegend, 100206) validated by the company (https://www.biolegend.com/en-us/products/pe-anti-mouse-cd3-antibody-47) and users (cited 104 times)
anti-CD4-APC (Biolegend, 100411) validated by the company (https://www.biolegend.com/en-us/products/apc-anti-mouse-cd4-antibody-245) and users (cited 83 times)
anti-CD8a-FITC (Biolegend, 100706) validated by the company (https://www.biolegend.com/en-us/products/fitc-anti-mouse-cd8a-antibody-153) and users (cited 28 times)
anti-IFNg-BV785 (Biolegend, 505838) validated by the company (https://www.biolegend.com/en-us/products/brilliant-violet-785-anti-mouse-ifn-gamma-antibody-7987) and users (cited 23 times)
anti-GZMb-AF647 (Biolegend, 515405) validated by the company (https://www.biolegend.com/en-us/products/alexa-fluor-647-anti-human-mouse-granzyme-b-antibody-6067) and users (cited 49 times)
anti-Tbet-BV711 (Bioloegend, 644819) validated by the company (https://www.biolegend.com/en-us/products/brilliant-violet-711-anti-t-bet-antibody-7952) and users (cited 8 times)
anti-IL-10-BV421 (Biolegend, 505022) validated by the company (https://www.biolegend.com/en-us/products/brilliant-violet-421-anti-mouse-il-10-antibody-7190) and users (cited 14 times)
anti-IL-12-PE (Biolegend, 505204) validated by the company (https://www.biolegend.com/en-us/products/pe-anti-mouse-il-12-il-23-p40-monomer-dimer-heterodimer-antibody-928) and users (cited 12 times)
Streptavidin-PE/Cy5 (Biolegend, 405205) validated by the company (https://www.biolegend.com/en-us/products/pe-cyanine5-streptavidin-1476) and users (cited 12 times)
Live/Dead-NearIR stain (Thermo, L10119) validated by the company (https://www.thermofisher.com/order/catalog/product/L34975)
DAPI (Biolegend, 422801) validated by the company (https://www.biolegend.com/en-us/products/dapi-4-6-diamidino-2-phenylindole-dilactate-8108) and users (cited 88 times)
anti-CRT-PE (Cell Signaling, 19780S) validated by the company (https://www.cellsignal.com/products/antibody-conjugates/calreticulin-d3e6-xp-rabbit-mab-pe-conjugate/19780) and users (cited 1 time)
anti-CRT (Cell Signaling, 12238) validated by the company (https://www.cellsignal.com/products/primary-antibodies/calreticulin-d3e6-xp-rabbit-mab/12238?site-search-type=Products&N=4294956287&Ntt=12238%29&fromPage=plp&_requestid=2423722) and users (cited 4 times)
anti-HSP70-PE (Miltenyi, 130-105-549) validated by the company (https://www.miltenyibiotec.com/US-en/products/hsp70-antibody-anti-human-mouse-reafinity-rea349.html#pe:100-tests-in-200-ul) and users (cited 3 times)
anti-COX-2 (Cell Signaling, 12282S) validated by the company (https://www.cellsignal.com/products/primary-antibodies/cox2-d5h5-xp-rabbit-mab/12282) and users (cited 109 times)
anti-GAPDH (Santa Cruz biotechnology, SC-32233) validated by the company (https://www.scbt.com/p/gapdh-antibody-6c5) and users (cited 3668 times)
anti-CD3 (Abcam, ab16669) validated by the company (https://www.abcam.com/CD3-antibody-SP7-ab16669.html) and users (cited 449 times)
anti-CD11c(Cell Signaling Technologies, 97585) validated by the company (https://www.cellsignal.com/products/primary-antibodies/cd11c-d1v9y-rabbit-mab/97585) and users (cited 4 times)
anti-aSMA (Abcam, ab5694) validated by the company (https://www.abcam.com/alpha-smooth-muscle-actin-antibody-ab5694.html) and users (cited 2187 times)
anti-HMGB1 (Biolegend, 651402) validated by the company (https://www.biolegend.com/en-us/products/purified-anti-hmgb1-antibody-7483) and users (cited 5 times)
anti-mouse-HRP (Boster, BA1075) validated by the company (https://www.bosterbio.com/hrp-conjugated-goat-anti-mouse-igm-u-chain-specific-secondary-antibody-ba1075-boster.html) and users (cited 4 times)
anti-rabbit-HRP (Cell Signaling, 7074S) validated by the company (https://www.cellsignal.com/products/secondary-antibodies/anti-rabbit-igg-hrp-linked-antibody/7074) and users (cited 4155 times)

anti-CD16/32 antibody (Biolegend, 101320) validated by the company (https://www.biolegend.com/en-us/products/trustain-fcx-anti-mouse-cd16-32-antibody-5683) and users (cited 277 times)
anti-Mouse IgG H&L HRP polymer (Abcam, ab214879) validated by the company (https://www.abcam.com/goat-mouse-igg-hl-hrp-polymer-ab214879.html) and users (cited 8 times)
anti-Rabbit IgG H&L HRP polymer (Abcam, ab214879) validated by the company (https://www.abcam.com/goat-rabbit-igg-hl-hrp-polymer-ab214880.html) and users (cited 23 times)

Eukaryotic cell lines

Policy information about cell lines

Cell line source(s)	G69, G7 (generated in-house)
Authentication	Not authenticated
Mycoplasma contamination	G69 and G7 tested negative for mycoplasma
Commonly misidentified lines (See ICLAC register)	No cell lines used are commonly missidentified in this study

Animals and other organisms

Policy information about studies involving animals; ARRIVE guidelines recommended for reporting animal research

Laboratory animals	FVB/NJ male mice ranging between 6-8 weeks of age were used; all mice were maintained in ambient room temperature (22 +/- 2 oC) with humidity of 40%-70% and light/dark cycle of 12h/12h.
Wild animals	No wild animals were used
Field-collected samples	No field-collected samples were used
Ethics oversight	All experiments performed were in accordance with procedures approved by the Institutional Animal Care and Use Committee of Cedars-Sinai Medical Center.

Note that full information on the approval of the study protocol must also be provided in the manuscript.

Flow Cytometry

Plots

Confirm that:

- [x] The axis labels state the marker and fluorochrome used (e.g. CD4-FITC).
- [x] The axis scales are clearly visible. Include numbers along axes only for bottom left plot of group (a 'group' is an analysis of identical markers).
- [x] All plots are contour plots with outliers or pseudocolor plots.
- [x] A numerical value for number of cells or percentage (with statistics) is provided.

Methodology

Sample preparation	All immune cell samples (e.g., tumor and vdLN immune cells processed using ACK lysis buffer prior to immunophenotyping) were suspended in 50ul of anti-CD16/32 antibody (Biolegend, 101320) solution at a dilution of 1:200 for 10 min on ice prior to subsequent to antibody staining. Immunophenotype staining was performed with antibodies diluted to 1:200 (final concentration) in FACS buffer on ice for approximately 20 min. DAPI (Biolegend, 422801) or Live/Dead-NearIR stain (Thermo, L10119) was used to exclude dead cells during analysis. Cancer cell lines from in vitro experiments were stained with either anti-CRT-PE (Cell Signaling, 19780S) or anti-HSP70-PE (Miltenyi, 130-105-549), using a final concentration of 1:200. These cells were stained with DAPI at a final concentration of 3uM in FACS buffer.
Instrument	BD LSRFortessa, Cytek Northern Lights
Software	FlowJo V10.7.1
Cell population abundance	No sorting was performed
Gating strategy	Gating strategy (basic): all samples were gated on FSC/SSC and doublet discrimination was performed on FSC-H/FSC-A.<br>Gating strategy (BMDCs and vdLN DCs): samples were gated on CD45+/exclusion dye(neg), MHCII(pos)/CD11c(pos), and subsequently for CD40, CD86, IL-10, IL-12, IL-4 and H2-k<br>Gating strategy (CD3+ T cells): samples were gated on CD45+/exclusion dye(neg), CD3(pos), CD4(neg)/CD8(pos), and subsequently for Tbet, IFNg, and GZMb.

- [ ] Tick this box to confirm that a figure exemplifying the gating strategy is provided in the Supplementary Information.
